# High density genome wide genotyping-by-sequencing and association identifies common and low frequency SNPs, and novel candidate genes influencing cow milk traits

**DOI:** 10.1038/srep31109

**Published:** 2016-08-10

**Authors:** Eveline M. Ibeagha-Awemu, Sunday O. Peters, Kingsley A. Akwanji, Ikhide G. Imumorin, Xin Zhao

**Affiliations:** 1 Agriculture and Agri-Food Canada, Sherbrooke Research and Development Centre, 2000 Rue College, Sherbrooke, QC, J1M 0C8 Canada; 2 Department of Animal Science, Berry College, Mount Berry, GA 30149 USA; 3 Department of Animal Science, McGill University, 21,111 Lakeshore Road, Ste-Anne-De-Bellevue, QC, H9S 3V9 Canada; 4 Animal Genetics & Genomics Lab., International Programs, College of Agriculture & Life Sciences, Cornell University, Ithaca, NY 14853 USA

## Abstract

High-throughput sequencing technologies have increased the ability to detect sequence variations for complex trait improvement. A high throughput genome wide genotyping-by-sequencing (GBS) method was used to generate 515,787 single nucleotide polymorphisms (SNPs), from which 76,355 SNPs with call rates >85% and minor allele frequency ≥1.5% were used in genome wide association study (GWAS) of 44 milk traits in 1,246 Canadian Holstein cows. GWAS was accomplished with a mixed linear model procedure implementing the additive and dominant models. A strong signal within the centromeric region of bovine chromosome 14 was associated with test day fat percentage. Several SNPs were associated with eicosapentaenoic acid, docosapentaenoic acid, arachidonic acid, CLA:9c11t and gamma linolenic acid. Most of the significant SNPs for 44 traits studied are novel and located in intergenic regions or introns of genes. Novel potential candidate genes for milk traits or mammary gland functions include *ERCC6, TONSL, NPAS2, ACER3, ITGB4, GGT6, ACOX3, MECR, ADAM12, ACHE, LRRC14, FUK, NPRL3, EVL, SLCO3A1, PSMA4, FTO, ADCK5, PP1R16A and TEP1*. Our study further demonstrates the utility of the GBS approach for identifying population-specific SNPs for use in improvement of complex dairy traits.

Milk is an important source of nutrients in human nutrition all over the world. In particular, milk polyunsaturated fatty acids (PUFAs) like isomers of conjugated linoleic acid (CLA), arachidonic acid (AA, 20:4n-6), eicosapentaenoic acid (EPA, 20:5n-3) and docosahexaenoic acid (DHA, 22:6n-3) have known positive associations with a range of human health conditions like cardiovascular diseases, anticancer effects, antiadipogenic, antiatherogenic, antidiabetogenic and anti-inflammatory properties^1^,^2^,^3^. For these reasons, dairy producers are looking for ways to optimize milk beneficial components.

Genetic variability in the relative proportions of the various milk components (proteins, fats, individual fatty acids, lactose, milk urea nitrogen, etc.) exist between and among cattle breeds^4^,^5^, which is further influenced by nutrition and gene x environment interactions. This variability indicates the possibility of using genomic selection to improve milk traits^6^,^7^,^8^,^9^.

Advances in high-throughput sequencing technologies provide the ability to improve complex traits. Over the past two decades, sequence variation information has supported genome wide association (GWAS) and candidate gene studies of milk traits^10^,^11^,^12^,^13^,^14^,^15^,^16^,^17^,^18^,^19^. Use of genomic information is gaining wide application in livestock improvement schemes^20^,^21^ since genotype data and confirmed associations with trait is important to support informed decisions in livestock selection.

Out of 36,693 quantitative trait loci (QTL) for 492 traits archived in the cattle QTL data base, about 5,815 are QTLs for milk fat composition, 3,157 for milk protein composition, 1,324 for milk yield, 550 for fatty acid content and 1,246 for mastitis (CattleQTLdb, http://www.animalgenome.org/cgi-bin/QTLdb/BT/index, accessed on 27 November, 2015). These QTLs are spread on most bovine chromosomes but only a few of the causative genes have been identified. Furthermore, majority of reported associations involves common variants with minor allele frequencies (MAF) >5%, while the contributions of low frequency (MAF 0.5% to 5%) and rare (MAF<0.5%) variants remain relatively untapped. Moreover, reported associations with milk traits have uncovered few markers or genes that explain a huge portion of the variation in traits^13^,^22^ while the remaining portion of unexplained variation may be attributable to low frequency and rare variants and remain to be uncovered.

A deeper study of the bovine genome to quantify the contribution of low frequency SNP variants and novel positional candidate genes is necessary. Recently, whole genome sequencing of 234 bull genomes of the Holstein, Fleckvieh, Jersey and Angus breeds uncovered 28.3 million variants, including common and low frequency variants responsible for various conditions in cattle^23^. Low frequency variants are not represented in currently available low and high density genotyping chips, a situation now remedied by next generation sequencing technologies in humans^24^, maize^25^ and cattle^26^. Using genotyping-by-sequencing (GBS) on the Illumina platform, De Donato *et al*.^26^ successfully identified and genotyped 63,697 SNPs in 47 bovine samples from 7 breeds in which they uncovered more SNPs per bovine chromosome than represented on the Illumina Bovine50KSNP BeadChip. Furthermore, they demonstrated the cost effectiveness of GBS as a complementary tool to available genotyping chips and its potential application to bovine studies and to other species.

In this study, we applied GBS to 1,246 Canadian Holstein cows from 16 different herds in Quebec followed by GWAS and identified population specific SNP markers, novel SNPs, novel SNP associations and novel candidate genes for milk traits.

## Results

### Sequencing results, identified SNPs and classification

**Figure 1:**
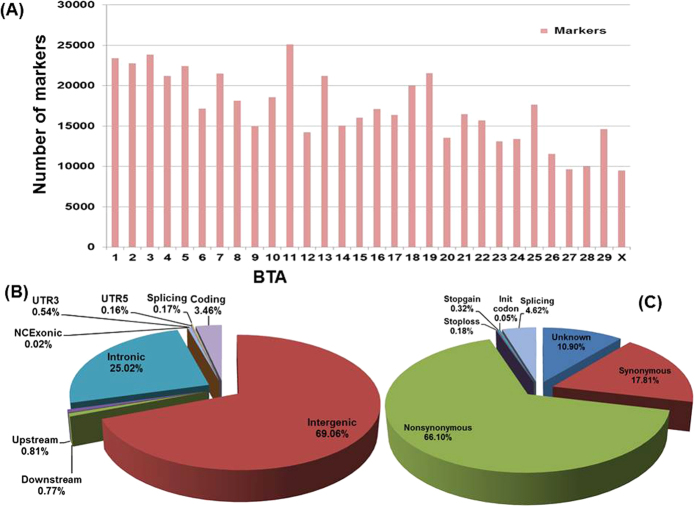
Distribution of identified markers by chromosomes (**A**), variant classification (**B**) and coding variant classes (**C**).

The method of GBS was used to analyse DNA samples from 1,246 Canadian Holstein cows on an Illumina HiSeq 2000 system. Sequencing generated a total of 3.7 billion reads. After initial quality check, 2.9 billion reads resulting in a total of 92.7 million unique tags were retained (Table S1). Unique tags were merged to a total of 14.6 million merged tags, of which 79.6% aligned to unique positions on the bovine genome (Btau 4.6.1), 10.5% aligned to multiple positions while 9.9% could not be aligned. By analyzing only tags that aligned to unique positions, a total of 515,787 SNPS were identified on all chromosomes. The highest number of SNPs were detected on BTA11 (25,093 SNPs) followed by BTA3 (23,860 SNPs) and the least number on BTAX (9,471 SNPs) (Fig. 1, Table S2). SNP variant classification indicated that majority of identified SNPs were located within intergenic regions (69%) of the genome followed by intronic regions of genes (25%) (Fig. 1, Table S2). Only 3.46% of SNPs are coding variants. About 4,280 SNPs located on 367 invalid transcripts were not classified. Further classification of coding SNPs indicated that 66% are non-synonymous, 18% are synonymous, 11% are unknown, 4.6% are splicing variants while SNPs at initiation codons, stop gain and stop loss constituted less than 1% (Fig. 1, Table S2). Majority of identified markers had MAF ≤1% (Figure S1). Only about 29% of identified markers are represented in dbSNP implying that 71% of identified variants are novel (Table S3a). These novel variants have been submitted to dbSNP and they have been assigned Submitted SNP (SS) numbers (Table S3b).

### Genotype imputation

Genotypes were imputated using Beagle v3.3.2^27^ to correct for missing genotypes in some samples. Principal component analysis (PCA) was used to assess population structure. Initial inconsistent PCA patterns in 3 herds (Figure S2) were resolved when call rate ≥80% and minor allele frequency (MAF) ≥1% filters were applied (Figure S3). Call rates were also improved post-imputation (Figure S4), with MAF generally unchanged.

### Results of significant genome wide association analysis

After genotype imputation, a total of 76,355 SNPs out of 515,787 with call rates >85%, accuracy of imputation score >50% and MAF ≥1.5% were retained and used in GWAS. Also excluded from GWAS were genotypes that deviated significantly from Hardy-Weinberg Equilibrium assumptions. Results of GWAS and Benjamini-Hochberg (BH) false discovery rate (FDR) correction (p-values BH FDR <0.1) are listed in Tables S4a to e, S5a to d, S6a to b and S7a to h. Only associations with corrected p-values BH FDR <0.1 were considered to be of genome wide significance in this study.

### Significant genome wide associations between markers and milk component traits

Markers were tested for significant associations with test day fat% (TFP), test day fat yield (TFY), 305 day fat yield (305dFY), test day protein% (TPP), test day protein yield (TPY), 305 day protein yield (305dPY), test-day milk yield (TMY), 305 day milk yield (305dMY), lactose% (LP), milk urea nitrogen (MUN) and milk somatic cell counts (SCC) (Table 1). Significant GWAS results (p-value BH FDR <0.1) were recorded between 1 to 143 markers and 7 variables (TFP, TPP, 305dFY, TMY, LP, MUN and SCC).

**Figure 2:**
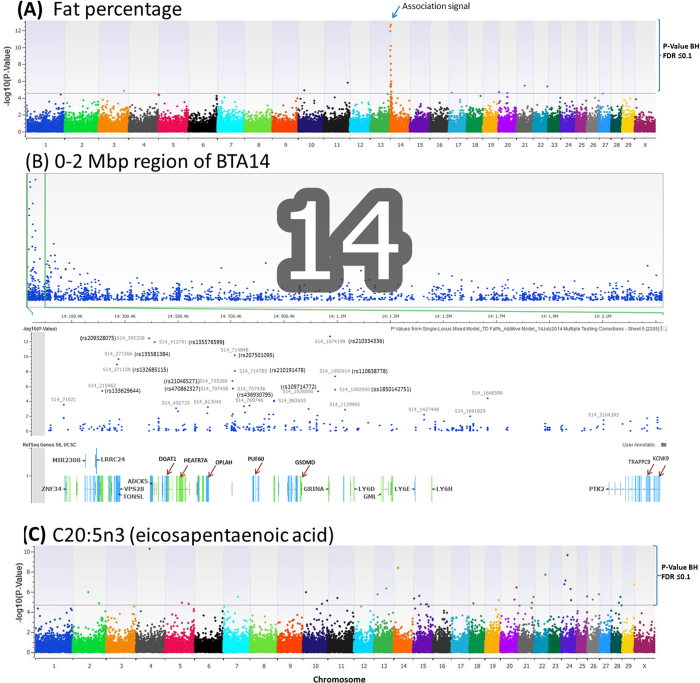
Manhatan plot of –log10(p-value) of genome wide SNP association results showing (**A**) a strong association signal at the centromeric region of BTA14 with test day fat percent (TFP); (**B**) expanded 0-2 Mbp region of BTA14 showing significant SNPs and corresponding genes and (**C**) significant SNP association with C20:5n3 (eicosapentaenoic acid, EPA). Thirty six and 56 significant (P-value BH FDR ≤ 0.1) genome wide SNP associations with respectively TFP and C20:5n3 are shown above the horizontal lines. Genome wide association analysis was done with implementation of the single-locus efficient mixed model (EMMA) association approach (Kang 2008) using 76,355 SNPs markers with minor allele frequencies ≥1.5%.

Thirty six markers (26 intergenic and 10 gene region variants) were significantly associated with TFP (Table S4a). In addition, a strong association signal was recorded between 20 markers in the centromeric region of BTA14 and TFP (Table 2, Fig. 2) out of which two (rs132685115, rs135581384) are located within *TONSL* gene and one each within *ADCK5* (rs135576599), *PP1R16A* (rs133629644) and *TRAPPC9* (rs207542860) genes. Only one coding region SNP (ss1850090958) within *TEP1* gene associated significantly with TFP and TPP.

Fifty three markers including 20 gene region SNPs were significantly associated (p-value BH FDR < 0.1) with TPP (Table S4b). Significant markers for TPP are spread over several chromosomes except BTA2, 12, 23, 26 and X (Table S4b). Two gene region SNPs are coding mutations (ss1850241810, ss1850090958) including a non-synonymous mutation (ss1850241810) within exon 5 of *P4HTM* gene.

In this study, the highest number of markers comprising 91 intergenic and 52 gene region SNPs were associated with SCC (Table S4c). Two variants each on *PLK1S1* (rs133818453, rs384381919) and *RILPL2* (ss1850180613, ss1850180614) genes were significantly associated with SCC and three intronic SNPs (rs459258791, rs470194324, rs211180730) have MAF above 16%. Interestingly, eight SNPs (rs462951569, ss1850157510, ss1850127088, rs445074791, ss1850272832, ss1850161426, rs384261616, ss1849973836) each explained about 4% or more of the variance in SCC.

Fewer significant associations were recorded between TMY, 305dFY, LP and MUN and studied markers (Table S4d,e). About 35 significantly associated SNPs with TFP, TPP, SCC, TMY and 305dFY, and having MAF ≥10% were considered the most commonly associated SNPs for these traits in this study (Table 3).

### Results of significant genome wide associations between markers and individual milk fatty acids

Several markers were significantly associated with two omega-3 fatty acids (C20:5n3, eicosapentaenoic acid, EPA and C22:5n3, docosapentaenoic acid, DPA), one omega-6 fatty acid (C20:4n6, arachidonic acid, AA), one CLA isomer (CLA:9c11t) and gamma linolenic acid (C18:3tcc) (Table S5a–d). Significant associations with EPA included 36 SNPs in intergenic regions and 20 markers within coding and regulatory regions (introns and 3′UTR) of 20 genes on 15 chromosomes (Table 4 and Table S5a). Seven of these significant associations were concordant by both additive and dominant models, 11 by the additive and two by the dominant models only. A non-synonymous coding SNP (p.Ala1424Pro) located within exon 21 (rs470755489) in the *ERCC6* gene with MAF of 11.2% was identified by the additive model to be significantly associated with EPA. Another non-synonymous coding SNP (ss1850036184) within exon 2 of *TTC38* gene (p.Ala16Pro) was significant for EPA by the additive model only.

SNPs in mostly intergenic regions were significantly associated with AA (C20:4n6) along with 25 markers on 25 genes (Table 4 and Table S5b). Only one coding synonymous SNP (ss1850101917) within exon 3 of the *TLX2* gene showed significant genome wide association with AA, whereas the highest phenotypic variance of 4.3% was explained by an intronic mutation (ss1850112671) within the *RABEPK* gene.

Only five markers (two within genes and three in intergenic regions) were significantly associated with DPA (C22:5n3) (Table 4 and Table S5c). An intergenic SNP (rs466855972) with the highest MAF (14%) is located within 50 Kbp of the *SMOC2* gene on chromosome 9. This mutation also explained the most variance (3.6%) in DPA. A silent mutation within exon 4 of the *GNLY* gene was the only coding SNP (ss1850106318) with significant association with DPA. One intergenic SNP on BTA1 (ss1849964962) with a MAF of 4% associated significantly with CLA:9c11t (Table 4 and Table S5d). Similarly, only one intergenic variant on BTAX (ss1850306238) reached genome wide significance with gamma linolenic acid (C18:3tcc) (Table 4 and Table S5d).

Palmitoleic acid (C16:1) out of seven monounsaturated fatty acids (MUFAs) (Table 1) was associated with one intergenic SNP on chromosome 26 (rs110405215) and oleic acid (C18:1n9c) was associated with three SNPs (ss1850063824, rs135581384 and rs41855732) on three different chromosomes (Table S6a). Nine SNPs on 8 different chromosomes associated significantly with total MUFA including one synonymous coding region SNP (ss1850271826) within exon 2 of the *ACHE* gene and two intronic SNPs in the *TACR3* (rs440980096) and ITGB4 (rs109739948) genes (Table S6b). Furthermore, SNP rs41855732 associated significantly with both oleic acid and total MUFA.

Significant marker associations with butyric acid (C4:0) are shown in Table S7a. Out of 116 associations, 81 markers are found in intergenic regions while 35 are found within gene regions. Majority of gene region associated variants are intronic (29) followed by three coding SNPs (rs457014340, rs456001743 and ss1850186363), two 3′UTR SNPs (rs480031082 and ss1850158549) and one splice variant (ss1850027498). Rs457014340 detected by both the additive and dominant models is located within exon 4 of the *VPS37C* gene (p.Ser115Ala, c.343T>G). rs456001743 and ss1850186363 were detected only by the additive model and located in exon 6 of the *COMMD4* gene (p.His102Pro, c.305A>C) and on exon 19 of the *FUK* gene (p.Gln855Glu, c.2563C>G), respectively.

Results of GWAS analysis for caproic acid (C6:0) are shown in Table S7b. One non-synonymous coding SNP (rs136905662, p.Gly265Val, c.794G>T) on F7 gene and two intronic variants on *PSMA4* (rs207776812) and *BICD2* (rs463987848) genes (have MAF of 10%) associated significantly with C6:0. Further intronic SNPs within *EVL* (rs467244058 and rs432423874), *FTO* (rs133525188 and rs381581176) and *MACROD*1 (ss1850302054 and rs451632156) genes associated significantly with C6:0. Significantly associated SNPs with C6:0 and having MAF ≥1.5% are shown in Table S7b. Furthermore, 7 markers (rs458879791, rs447857210, ss1850048597, ss1850251288, rs451632156, ss1850074571, rs469668684) out of 69 significant gene region SNPs explained the highest phenotypic variances in C6:0, ranging from 3.2% to 6.9%.

Caprylic acid (C8:0) was significantly associated with 119 SNPs including 81 intergenic and 38 gene region SNPs (including 4 non-synonymous coding SNPs) (Table S7c). Two intronic SNPs (ss1850302054 and rs451632156) within *MACROD1* gene were found to be significantly associated with C6:0 and C8:0. Five SNPs (rs451632156, rs458879791, rs447857210, ss1850251288, rs110927574) explained relatively high proportions of the variance in C8:0, ranging from 31% to 5.5%.

Significant associations were detected between five SNPs (ss1850128726, ss1850120683, ss1850133655, ss1850196022 and ss1850063824) and C14:0, and between two SNPs each and C11:0 (rs43649533, ss1850043060), C15:0 (rs382773693, ss1850118039) and C17:0 (rs385021638, ss1850255586) (Table S7d). Ss1850196022 (p.His30Pro, c.89A>C) is a non-synonymous coding SNP within exon 1 of *CYP2S1* gene.

The most significant GWAS results were recorded for tridecylic acid (C13:0), with 707 markers out of 76,355 (Table S7e). Out of this number, 483 are intergenic SNPs while 224 including 27 coding variants (21 are non-synonymous) are gene region SNPs. Furthermore, one coding SNP (rs471212184) in exon 9 of the *TARBP2* gene is a stop loss mutation (p.*367Glyext*64, c.1099T>G). Although most SNPs associated with C13:0 had MAF below 10%, 5 gene region SNPs had MAF ranging from 10.98% to 20.91 (Table S7e) while 16 intergenic variants had MAF above 10%. It should be noted that 5 intronic SNPs within *NPAS2* gene associated with C13:0. Similarly, two SNPs in each of *GRB10, CSGALNACT1, XPNPEP1, MAD1L1* and *CLSTN2* genes attained genome wide significance with C13:0. Furthermore, two SNPs within microRNA genes, (rs440208182 on *MIR130B/MIR301B* and rs480300366 on *MIR3596/MIRLET7B*) associated with C13:0.

Tricosanoic acid (C23:0) and lignoceric acid (C24:0) long chain saturated fatty acids (SFAs) associated significantly with several SNP markers while no associations were recorded for C20:0 and C22:0 (Table S7f,g). Out of 40 significant markers (30 intergenic, 9 intronic and one 5′UTR) for C23:0, 7 intergenic SNPs had MAF of 10% and above. Furthermore, 12 intergenic variants and 3 intronic variants each accounted for over 3% of phenotypic variance in C23:0. Significant associations for C24:0 included 164 intergenic and 80 gene region SNPs (Table S7g). Three intronic variants (ss1850076897, rs42155039 and ss1850107740) on *MYT1L, MARCH8* and *ANTXR1* genes, respectively, had MAF of respectively of 29.4%, 22.6% and 18.6%. Seven out of nine coding SNPs are non-synonymous (Table S7g). Two intronic SNPs within *GMDS* (ss1850253438) and *ACPP* (ss1849970456) genes explained the highest proportion of variance in C24:0 at 7.3% and 6.3% respectively. Only 11 SNPS on 10 different chromosomes, including one coding SNP on *ACHE* (ss1850271826) gene and one intronic SNP each on *TONSL* (rs135581384) and *TACR3* (rs440980096) genes associated significantly with milk total SFA (Table S7h).

One or more SNPs within the same gene associated significantly with one or several traits (Table S8). In some cases, one SNP associated significantly with two or more fatty acids (Table S9). For example, one SNP each within *ACOX3*, *PPP2R4* and *GGT6* genes associated significantly with C4:0, C6:0 and C8:0, 5 SNPs within NPAS2 gene associated significantly with C13:0 while five variants within *EVL* gene associated significantly with one or more fatty acids. Several markers within seven regions (1 Mbp to 3 Mbp) denoted association hotspots, on different chromosomes associated significantly with several fatty acids (Table 5). About 81 significantly associated variants with individual milk fatty acids with MAF ≥10% were considered commonly associated SNPs with these traits, in this study (Table 6).

## Discussion

We have demonstrated in this study that genotyping-by-sequencing (GBS) technique is a useful approach to detect population-specific SNPs influencing milk traits in Canadian Holstein cows by identifying 515,787 SNPs in 1,246 animals using the Illumina HiSeq platform. This technique was first developed to support genetic diversity studies in plants^25^ and tested on cattle samples at a small scale by De Donato *et al*.^26^ and has now been extended to identify SNPs in a much larger sample size followed by GWAS.

GBS allows a higher level of ascertainment of the genetic variation within a given population than current genotyping arrays and at a cheaper cost (about a third of the cost of using proprietary Illumina BovineSNP50K array). About 71% of detected SNPs in this study are novel, most of which may be population-specific, will increase our knowledge of genomic variation in Canadian Holstein cows available for dairy improvement. It should be noted that it is best to apply results of marker trait association information in populations were such associations were identified. The GBS technique may therefore compliment available genotyping arrays for detection of novel and known SNPS within specific populations.

The preponderance of MAF of <5% in this study is not unusual because whole genome sequencing of 234 bulls representing Holstein, Fleckvieh, Jersey and Angus breeds and deep sequencing of human genomes from different racial backgrounds indicate that rare (MAF below 0.5%) and low (MAF 0.5% to 5%) frequency variants greatly outnumber common variants^23^,^24^. Furthermore, rare and low frequency variants have been shown to explain part of the phenotypic variation in some human diseases^28^. Our findings showed that majority of significant SNPs for milk traits are found within non-coding regions of genes and intergenic regions of the genome and is supported by many recent GWAS and candidate gene studies on milk traits^14^,^29^,^30^. In humans, it has been reported that over 80% of disease associated variants fall outside protein coding regions of genes^31^, further strengthening the contribution of non-coding SNPs and intergenic region SNPs to complex traits, and supports their inclusion in GWAS. Our data further strengthens the notion that previously considered junk regions of the genome now harbor mutations that drive gene expression and affect the outcome of economically important traits.

As complex quantitative traits are controlled by numerous genes with small effects^22^,^32^, milk traits were associated mostly with SNPs with small effects. This study confirmed a strong signal for TFP in the centromeric region of BTA14 previously reported for milk fat yield, fat%, protein yield and protein%^18^,^22^,^29^,^30^,^32^,^33^,^34^,^35^,^36^. This peak region (0–2 Mbp, Fig. 2) lies within the same chromosomal region as the *DGAT1* gene whose effect on milk production traits has been confirmed in numerous breeds around the globe^11^,^17^,^22^,^33^,^37^,^38^,^39^. Smaraqdov^38^ has proposed the use of the *DGAT1* K232A mutation as a golden standard in gene sets used in the comparison of effects on milk productivity. However, the pleiotropic effect of the K232A polymorphism on genes related to cell growth, proliferation, development, tissue remodeling, cell signaling and immune system response has led to the argument that the expression pattern of genes carrying the K232A mutations reflect counter mechanisms of mammary gland tissue response to changes in milk fatty acid concentration and/or composition^40^. Streit *et al*.^39^ showed evidence for a major *DGAT1* gene by polygene interaction effects for milk fat and protein percentage in German Holstein cattle while Bennewitz *et al*.^33^ reported that *the DGAT1* K232A mutation is not solely responsible for all the genetic variation for milk, fat and protein yield and fat and protein percentages at the centromeric region of BTA14. Our data has uncovered more SNPs that contribute to the genetic variation in TFP in the centromeric region of bovine BTA14. Although 15 out of the 20 strong signal variants for TFP on BTA14 are located within intergenic regions, 5 are located within the intronic regions of four genes (*ADCK5, TONSL, PPP1R16A* and *TRAPPC9*) (Table 2). *TONSL, ADCK5 and PP1R16A* are among genes identified in a study that assessed the gene content of the chromosomal regions flanking the *DGAT1* gene as a basis for future linkage disequilibrium studies with aim to determine whether neighboring genes to *DGAT1* are associated with variation in milk fat percentage^41^. The two mutations of *TONSL* (rs132685115 and rs135581384) had MAF of respectively 27% and 26% and explained 9.5% of the variation in TFP in this study and may be considered potential candidate markers for milk fat%. An intergenic variant (rs210334336) positioned at 0.7 Mbp upstream of *DGAT1* gene has a MAF of 29% and explained the highest proportion of variance (6.6%) in TFP in this study. The high proportion of variance (72.85.4%) in TFP explained by the significant SNPs (20 of them) within the centromeric region of BTA14 in this study supports the notion that *DGAT1* gene is not solely responsible for the variation in milk fat% in this region. Other SNPs within the centromeric region of BTA14 significantly influenced other traits in this study. These include intergenic SNPs (rs109818540, rs109072495 and rs110566728) and one coding SNP in *LRRC14* gene (rs439245899, c.500T>G, p.Val167Gly) significantly influenced C13:0 while two intergenic SNPs, rs110892754 and rs381071867, significantly associated with 305dFY and LP, respectively. In a recent GWAS utilizing the Illumina BovineSNP50 BeadChip for milk production traits in Chinese Holstein population, 92.3% (60 out of 65) of genome-wise significant variants for milk fat percentage were located within a 6.2 Mbp region (0.05–6.25 Mbp) of BTA 14^29^ further supporting our findings. The only non-synonymous coding SNP (ss1850090958) that showed genome wide significance with TFP and TPP in this study is located within exon 20 of the *TEP1* gene (p.Tyr1006Ser, c.3017A>C) and the affected amino acid lies within a region of unknown function of the protein.

Five significant associations for TPP (rs455358874, rs134756756, rs137597165, ss1850220972 and ss1850220213) are located on BTA20, mostly in the vicinity of reported QTLs for TPP^29^,^42^,^43^. Three further associated SNPs for TPP (ss1850047119, rs379699027, rs133974370) on BTA6 occur within a region (118 Mbp to 120 Mbp) where significant QTLs for protein percentage have been reported^34^,^44^ and could be contributing factors to these QTLs.

Many significant associations (91 intergenic and 52 gene region variants) were recorded for SCC. SCC is routinely monitored in dairy herds as an indirect measure of bovine mammary gland health. Mastitis, the most important disease of dairy cows is under the control of numerous factors including genetics, indicating that many genes and gene pathways spread over the entire genome may contribute to the genetic variance in milk SCC. The 52 associated SNPs with SCC are located either in the intronic or exonic regions of 48 genes. Many of these genes (e.g. *RASA3, TPST1, JDP2, PTPN22, CAMKK1, TNR, IGGL1, CDH15, CHD23, NGEF, ANKRD27, SBF2, TXNDC5, RRM3, CHST8, ADAM12*) with immune functions, are located in disease related pathways or are implicated in disease progression. Furthermore, several significantly associated SNPs with SCC in this study lie within reported QTL regions for SCC and somatic cell score^45^.

SNPs associated with one or more PUFAs were found on all chromosomes, except BTA 8, with 5 or more associations on BTA 1, 5, 7, 10, 11, 15, 19, 21, 24 and 28 (Table S5a to S5d). Only a few studies on candidate gene associations and significant QTLs for individual or total milk PUFAs have been reported in cattle^10^,^14^,^46^,^47^,^48^ and our study is the first to detect significant SNPs associated with milk EPA (C20:5n3), AA and DPA in genes without prior associations with fatty acid biosynthesis or uptake. Three SNPs (ss1850294609, rs470755489 and rs471314510) within a 10 Mbp region (33.4 Mbp to 43.5Mbp) of BTA28 associated significantly with EPA. Rs470755489 (p.Ala1424Pro) is a non-synonymous SNP within exon 21 of the *ERCC6* gene suggesting *ERCC6* as a likely novel candidate gene for milk EPA. Another potential candidate gene harboring rs439293424 for milk EPA is *ACER3* implicated in sphingolipid metabolism pathway. SNP rs439293424 (on *KCNJ1* gene) and ss1850305614 (on *LSP1* gene) are located within a chromosomal region harboring *FADS1* and *FADS2* genes, with well-defined roles in the synthesis of PUFAs. SNPs within *FADS1* and *FADS2* were recently demonstrated to associate significantly with C20:3n6, C20:4n6 and C20:5n3 in bovine milk^14^. A 28 Mbp region of BTA 24 (12.82 Mbp to 40.82 Mbp) harbored 8 SNPs (ss1850256121, ss1850256332, ss1850256353, rs385515058, rs381067250, rs209502433, rs445435952, rs109764724) significantly associated with C20:5n3 and together explained 25.23% of the variation in C20:5n3. There are only two reports of significant QTLs for milk fat% and milk fat yield in this region^49^,^50^.

Two SNPs (ss1850063824 and rs41855732) were associated with variation in both oleic acid and total MUFA. Oleic acid is the most abundant MUFA and obviously contributed the most to total MUFA. The intergenic ss1850063824 SNP is mapped close to two genes in the solute carrier family (*SLC7A4* and *SLC25A1*) as well as *MAN2A1* implicated in two KEGG pathways, metabolic and n-glycan biosynthesis pathways. Recently, Nafikov *et al*.^51^ reported a QTL for oleic acid on BTA 7 and suggested a gene in the solute carrier family (*SLC27A6*) as the potential candidate. An intronic SNP (rs109739948) in *ITGB4* gene that associated significantly with C18:1n9c occurs within the region (26.5–57.7 Mbp) of a reported QTL for milk C18:1n9c percentage^52^. *ITGB4* gene could be a candidate for oleic acid. A SNP in the *TONSL* gene (rs135581384) with a high MAF significantly associated with both C18:1n9c and TFP and may be a candidate SNP for these traits. Only one coding synonymous variant (ss1850271826) within *ACHE* gene with roles in metabolism and glycerophospholipid biosynthesis and metabolism pathways were significantly associated with total MUFA.

More significant associations were recorded for six SFAs (C4:0, C6:0, C8:0, C13:0, C23:0 and C24:0) compared to 16 SFAs studied. The most significantly associated SNP to C4:0, rs458879791, also associated with C6:0 and C8:0 and occurs in the intronic region of the *GGT6* gene with roles in glutathione metabolism^53^. This SNP explained 4.8%, 6.9% and 5.5% of the variation in C4:0, C6:0 and C8:0, respectively and is considered a potential candidate gene for these traits even though it has a low MAF of 1.5%. Furthermore, rs458879791 may be localized in the same region as a previously reported QTL on BTA19 for C6:0 and C8:0^51^. Another SNP (ss1850048597) that associated significantly with C4:0, C6:0 and C8:0 occur in the intronic region of *ACOX3* gene with documented roles in the biosynthesis of SFAs and fatty acid oxidation^54^. A *MECR* gene SNP (ss1849987546) significantly influenced C4:0. *MECR* is involved in catalyzing the NADPH-dependent reduction of trans-2-enoyl thioesters and generating saturated acyl-groups^55^. SNPs in *ACSL3* (ss1849985469) and *FABP3 (*ss1849987006) genes with well-defined roles in fatty acid biosynthesis^56^,^57^ on BTA2 were significantly associated with variation in C13:0 and C6:0, respectively. A SNP (rs379603734) on *ADAM12* gene implicated in breast cancer^58^ associated significantly with C4:0 and C13:0, and responsible for 2% of phenotypic variation. Three SNPs including a coding variant (ss1850186363, p.Gln855Glu, MAF of 3.8%) in exon 19 of *FUK* associated significantly with C4:0 and with a role in KEGG’s fructose and mannose metabolism pathway, may not be excluded from mammary lipogenesis.

Mutations on *NPRL3* gene (ss1850263261 [p.Val394Gly] and ss1850263264) with significant associations with respectively C4:0 and C24:0 occurs within a region of a QTL for milk palmitic acid (C16:0)^59^ making it a potential candidate gene for milk fatty acid traits. Numerous SNP associations and QTLs exist on BTA21 for milk traits in dairy cattle^19^,^46^,^50^,^60^ and this study has identified SNP associations with fatty acid traits in *EVL* (rs432423874, rs209872748, rs467244058, rs381368835 and rs454925079)*, SLCO3A1* (rs434552481 and rs209897920) and *PSMA4* (rs207776812) genes making them potential candidate genes for the reported QTLs. Neighboring SNPs (rs133525188 and rs381581176) occurring with the same MAF (4.2%) on the *FTO* gene influenced C6:0 significantly thus supporting a previous report on the impact of two causative mutations in the *FTO* gene with a functional effect on milk fat and protein yield in Holstein dairy cattle^61^. *MACROD1* and five other genes (*SLC22A8*, *NRXN2, EHD1, DKFZP761E198* and *C29H11orf68*) harboring variants with significant associations with C6:0, C8:0 and C13:0 are located within an association hotspot region (3 Mbp, from 42815829 to 45731092 Mbp) of BTA29 (Table 5). Previous reports of significant QTLs for milk and protein yield and for milk fat and protein percentages within this region of BTA29^36^,^43^ suggest that associated SNPs in this study could be contributing factors to the phenotypic variance in these traits. Two or more SNPs in 7 genes including 5 in *NPAS2* gene were observed to significantly associate with C13:0 in this study (Table S7e). SNPs in *NPAS2* gene are located within a reported QTL region for milk fat yield on BTA11^42^. In addition, two *NPAS2* SNPs (rs211557881 and rs208606161) explained about 7% of the variation in C13:0. The presence of *NPAS2* amongst genes of human REACTOME’s fatty acid, triacylglycerol and ketone body metabolism pathway suggest a similar role for this gene in bovine.

## Conclusion

Our study used GBS method to identify 515,787 SNPS in Canadian Holstein cows. Most SNPs were localized in intergenic regions followed by intronic regions of genes further emphasizing the contribution of non-coding and intergenic region variants in defining phenotypes and supports their inclusion in GWAS. Only about 29% of identified SNPs are present in dbSNP, while 71% are novel. Association of 76,355 markers with 44 milk traits identified novel genomic regions associated with milk traits. Most associated SNPs were located in intergenic regions followed by intronic regions of genes. Twenty markers within the centromeric region of bovine chromosome 14 showed strong association with TFP. Several SNPs were significantly associated with two omega-3 fatty acids (C20:5n3 [EPA] and C22:5n3 [DPA]), one omega-6 fatty acid (C20:4n6, AA), one CLA isomer (CLA:9c11t) and gamma linolenic acid (C18:3tcc). Several potential candidate genes uncovered for milk traits or mammary gland functions include *ERCC6, TONSL, NPAS2, ACER3, ITGB4, GGT6, ACOX3, MECR, ADAM12, ACHE, LRRC14, FUK, NPRL3, EVL, SLCO3A1, PSMA4, FTO, ADCK5, PP1R16A* and *TEP1*. Our study further demonstrated the utility of the GBS technique for identifying population-specific SNPs for use in improvement breeding of complex dairy traits.

## Methods

### Animal ethics

Animal use procedures and protocols were according to the national codes of practice for the care and handling of farm animals (http://www.nfacc.ca/codes-of-practice) and approved by the animal care committee of McGill University.

### Animals and milk sampling

About 1246 Canadian Holstein dairy cows enrolled in the dairy production center of expertise for Quebec and the Atlantic Provinces, Valacta (www.valacta.com) were used for this study. Cows were drawn from 16 herds from the province of Quebec with an average of 98 animals per herd. Cows were in mid-lactation and their parities ranged from one to five. Animal management by participating farms were according to standard procedures. Fifty mL of milk was collected from each animal during the morning milking and a portion of it (about 10 mL) was used to analyse for milk components while 40 mL was separated into fat and milk somatic cells by centrifugation (12000 × g at 4 °C for 30 min) immediately upon arrival at the laboratory. The fat portion was used for fatty acid profile analysis while DNA was isolated from milk somatic cells. Milk sampling was coordinated by Valacta.

### Analysis of milk components

The contents of milk components including test day milk yield, fat and protein yields, lactose and milk urea nitrogen were determined with MilkoScan FT 6000 Series mid-range infrared Fourier Transform Infra-Red (FTIR) based spectrometers, and the somatic cell counts were determined by means of Fossomatic flow cytometric cell counter at VALACTA (Ste-Anne de Bellevue, QC, www.valacta.com). Test day milk fat and protein yields were determined by multiplying the respective percentages with the total test day milk production. Entire lactation production values (305-d total milk production, 305-d milk fat yield and milk protein yield) were obtained by adding together monthly values covering the entire lactation period for each cow.

### Fatty acid profile analysis

Fatty acid methyl esters (FAME) for fatty acid profile analysis were prepared according to the procedure of O’Fallon *et al*.^62^. FAME were separated into different fatty acid isomers by capillary gas chromatography on a Varian CP-3900 gas chromatograph equipped with a Varian CP-8400 auto-sampler and auto-injector, column oven and a flame ionization detector (Varian Inc., Walnut Creek, CA, USA) according to O’Fallon *et al*.^62^. Individual FAME peaks were identified by comparison of retention times with FAME standards (GLC No. 463 and No. UC-59-M, Nu-Chek Prep Inc., Elysian, MN, USA). Agilent Technologies Chemstation (B.04.03) software was used for data analysis.

### DNA isolation

Genomic DNA from milk somatic cells was isolated using NucleoSpin^®^ Blood QuickPure kit (MJS Biolynx, Ontario, Canada) with some modifications as described in Ibeagha-Awemu *et al*.^14^. The concentration of purified DNA was measured with NanoDrop^®^ spectrophotometer (NanoDrop Technologies, Inc., Wilmington, DE, USA).

### Genotyping-by-sequencing (GBS)

GBS libraries were prepared and analyzed at the Institute for Genomic Diversity, Cornell University (IGD), according to Elshire *et al*.^25^ with modifications according to De Donato *et al*.^26^. To reduce genome complexity, samples were initially digested with *PstI* enzyme^26^. Libraries were created with 1246 unique barcodes. Ninety-six multiplexed libraries (includes controls) per lane (total of 15 lanes) were subjected to single end 100 bp sequencing on an Illumina HiSeq 2000 system (Illumina Inc., San Diego, CA, USA).

### GBS bioinformatics

**Figure 3:**
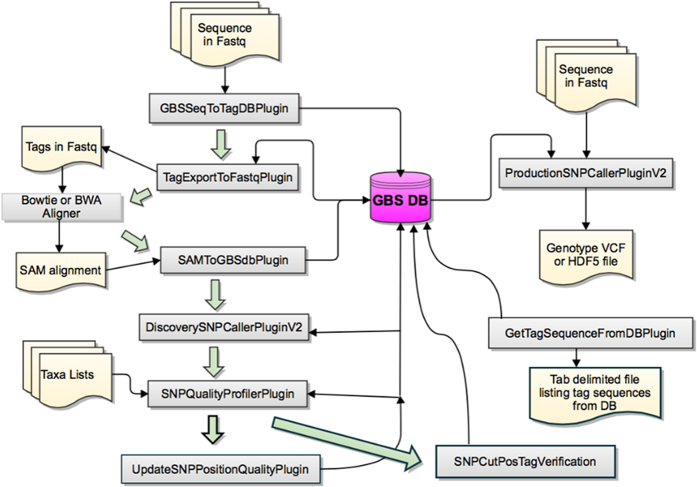
Genotyping-by-sequencing sequence data analysis pipe line (https://bitbucket.org/tasseladmin/tassel-5-source/wiki/Tassel5GBSv2Pipeline).

The GBS analysis pipeline (Fig. 3) implemented in Tassel Version: 3.0.139^63^ (date: November 8, 2012) was used to process raw Illumina DNA sequence data and to call SNPs. The GBS pipeline options used are listed in Table S10. An overview of GBS bioinformatics and the GBS pipeline can be found at http://www.maizegenetics.net/#!tassel/c17q9. Tags were aligned to the cow reference genome, Btau_4.6.1/bosTau7 assembly using BWA version 0.6.1-r104.

### SNP analysis

VCF tools v0.1.8 (http://vcftools.sourceforge.net/)^64^ was used to summarize data, filter data and to generate input files for PLINK^65^, which were used for multidimensional scaling (MDS). Analyses were visualized using basic plotting functions in R version 2.15.0 (https://www.r-project.org/).

### Genotype imputation

Internal imputation with Beagle v3.3.2 software was done to correct for missing genotypes at some marker sites in some samples and also to increase overall data call rates. Beagle was run with default parameters. The Beagle utility program “gprobs2beagle.jar” was used to make genotype calls based on a probability threshold of 0.95. Any subject/marker combination where the probability of the most likely genotype was less than 0.95 was assigned a missing genotype in the output and was not phased. The Beagle utility program “gprobsmetrics.jar” was used to compute several per-marker metrics including minor allele frequency and allelic R^2 values among other metrics.

### Genome wide association analysis

GWAS was accomplished with the single-locus mixed linear model procedure implemented in Golden Helix SVS v8.1.1 software (Golden Helix, Inc, Bozeman, MT, USA, www.goldenhelix.com). Specifically, the efficient mixed model association (EMMA) approach^66^ was used to directly estimate the variance components σ^2^_g_ and σ^2^_e_, reducing the problem to a maximization search in just one direction. To correct for population structure in the absence of pedigree data, a kinship matrix was computed once using all markers. The kinship matrix was then used to solve the EMMA equation for every marker. The EMMA procedure and equations have been described in details in SNP & Variation Suite Manual Release 8.4.3 (Golden Helix, Inc.) and in Kang *et al*.^66^ and summarized below:

The genotypes to phenotype association was done by testing the hypothesis 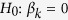 for each *m* loci one at a time, on the basis of the model (1)


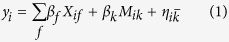


where *M*_*ik*_ is the minor allele count of marker *k* for individual *i*, *β*_*k*_ is a fixed effect size of marker *k*, and 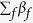 are other fixed effects of parity and herd. The error term 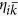 is (2)


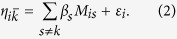


Assuming the 1246 Canadian Holstein dairy cows were unrelated and there was no dependence across the genotypes, the 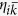 values will be independently and identically distributed (i.i.d.), and thus simple linear regressions will make appropriate inferences for the *k* values of *β*.

However, the variance of the first term of 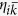 actually comes closer to being proportional to a matrix of the relatedness or kinship between samples. Thus (3),


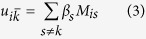


which reduces the equation *y*_*i*_ to the mixed-model equation (4)


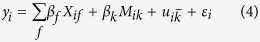


Both the additive and dominant models were used in GWAS. Under the additive model, testing is designed specifically to reveal associations which depend additively based on the allele classification. When alleles are classified according to frequencies, the associations will depend additively on the minor allele, where having two minor alleles (DD) rather than having no minor alleles (dd) is twice as likely to affect the outcome in a certain direction as is having just one minor allele (Dd) rather than no minor alleles (dd) (SVS Manual release 8.4.3, www.goldenhelix.com). Under the dominant model, allele classification according to frequency specifically tests the association of having at least one minor allele D (either Dd or DD) versus not having it at all (dd) (SVS Manual release 8.4.3). Both models were used in GWAS in this study to enable the capture of most existing associations. The Benjamini-Hochberg (BH) false discovery rate (FDR) correction was applied to raw p-values and genome wide significance was declared at P-Value BH FDR <0.1.

## Additional Information

**How to cite this article**: Ibeagha-Awemu, E. M. *et al*. High density genome wide genotyping-by-sequencing and association identifies common and low frequency SNPs, and novel candidate genes influencing cow milk traits. *Sci. Rep*. **6**, 31109; doi: 10.1038/srep31109 (2016).

## Supplementary Material

Supplementary Information

Supplementary Table S3

Supplementary Table S4

Supplementary Table S5

Supplementary Table S6

Supplementary Table S7

## Figures and Tables

**Table 1:** Studied milk component and fatty acid traits and their mean values (±standard error [SE]) across 16 herds.

Milk component traits	Acronym	Mean	±SE
Test day fat %	TFP	3.825	0.021
Test day fat yield (kg)	TFY	1.250	0.010
Actual 305 day fat yield (kg)	305dFY	395.227	1.962
Test day protein %	TPP	3.254	0.014
Test day protein yield (kg)	TPY	1.064	0.008
Actual 305 day protein yield (kg)	305dPY	335.009	1.544
Test day milk yield (kg)	TMY	32.812	0.271
Actual 305 day milk yield (kg)	305dMY	10380.22	51.664
Milk lactose (%)	LP	4.512	0.007
Milk urea nitrogen (mg/dl)	MUN	8.676	0.088
Milk somatic cell counts (x1000cells/ml)	SCC	269.880	16.443
**Fatty acid Traits**	**Common name**	**Mean**	**±SE**
Saturated Fatty acids (SFA)			
C4:0	Butyric acid	0.828	0.0043
C6:0	Caproic acid	1.0313	0.0048
C8:0	Caprylic acid	0.9106	0.0043
C10:0	Capric acid	2.6744	0.014
C11:0	Undecanoic acid	0.229	0.0015
C12:0	Lauric acid	3.4929	0.0193
C13:0	Tridecylic acid	0.4505	0.0083
C14:0	Myristic acid	12.298	0.0438
C15:0	Pentadecylic acid	1.1271	0.0074
C16:0	Palmitic acid	34.44	0.1023
C17:0	Margaric acid	0.6507	0.002
C18:0	Stearic acid	9.8168	0.0595
C20:0	Arachidic acid	0.1214	0.0009
C22:0	Behenic acid	0.0543	0.0004
C23:0	Tricosanoic acid	0.0313	0.0013
C24:0	Lignoceric acid	0.0417	0.0009
Total SFA	—	68.185	0.1186
Monounsaturated fatty acids (MUFA)			
C14:1 (C14:1*cis*-9)	Myristoleic acid	0.2968	0.0031
C14:1t (C14:1 *trans*-9)	Myristelaidic acid	1.0092	0.0083
C16:1 (C16:1 *cis*-9)	Palmitoleic acid	0.3067	0.0015
C16:1t (C16:1 *trans*-9)	Palmitelaidic acid	1.8038	0.011
C18:1n9c (C18:1 *cis*-9)	Oleic acid	20.216	0.1034
C18:1n9t (C18:1 *trans*-9)	Elaidic acid	0.2528	0.0028
C18:1n11t (C18:1 *trans* 11)	Trans vaccenic acid	1.0874	0.0125
Total MUFA	—	24.972	0.1085
Polyunsaturated fatty acids (PUFA)			
C18:2n6tt (C18:2 *trans*-9,12)	*Trans*-linoleic acid	0.1756	0.0014
C18:2n6cc (C18:2 *cis*-9-12)	Linoleic acid	1.8415	0.0107
C18:2n9c11t (C18:2 *cis*-9 *trans*-11)	*Cis*-9, *trans*-11 CLA	0.3904	0.0032
C18:2n10t12c (C18:2 *tran*s-10 *cis*-12)	*Trans*-10, *cis*-12 CLA	0.0159	0.0002
C18:3tcc (C18:*3 trans*-9 *cis* 12,15)	Gamma linolenic acid	0.1067	0.0006
C18:3n3 (C18: 3 *ci*s-9,12,15)	A Alpha linolenic acid	0.4175	0.0042
C20:3n6 (C20:3 *cis*- 8,11,14)	Dihomogamma linolenic acid	0.0917	0.0007
C20:4n6 (C20:4 *cis*-5,8,11,14)	Arachidonic acid	0.1302	0.0013
C20:5n3 (C20:5 *cis*- 5,8,11,14,17)	Eicosapentanoic acid	0.0338	0.0005
C22:5n3 (C22:5, *cis*-7,10,13,16,19)	Docosapentaenoic acid	0.1598	0.0051
Total PUFA	—	3.3275	0.0172

**Table 2:** Markers showing genome wide significant associations with test day fat percentage (TFP).

Marker	Chr	Position	RS or SS number	Classification	Gene/(nearby gene (s))^1^	Minor/Major Allele	P-Value^2^	P-Value^2^ BH FDR	Proportion Variance Explained	MAF^3^
Additive and dominant models										
S14_1074199	14	1074199	rs210334336	Intergenic	(GRINA, Them6)	T/G	2.09E-13 (1.53E-11)	1.4E-08 (3.8E-07)	0.0657	0.288
S14_392338	14	392338	rs209328075	Intergenic	(CPSF1)	T/C	3.72E-13 (6.99E-12)	1.4E-08 (2.6E-07)	0.0644	0.276
S14_412791	14	412791	rs135576599	Intronic	ADCK5	G/A	1.22E-12 (1.54E-14)	3E-08 (1.1E-09)	0.0616	0.259
S14_714848	14	714848	rs207501095	Intergenic	(MIR2309)	G/A	6.83E-11 (3.5E-10)	1.3E-06 (5.2E-06)	0.0522	0.307
S14_277266	14	277266	rs135581384	Intronic	TONSL	G/A	2.4E-10 (2.7E-11)	3.6E-06 (5E-07)	0.0493	0.257
S14_271158	14	271158	rs132685115	Intronic	TONSL	T/C	1.19E-09 (3.54E-08)	1.5E-05 (0.0004)	0.0455	0.271
S14_714785	14	714785	rs210191478	Intergenic	(MIR2309)	A/G	9.64E-09 (6.43E-09)	0.0001 (7.9E-05)	0.0406	0.315
S14_1092614	14	1092614	rs110638778	Intergenic	(GRINA, Them6)	A/G	5.09E-08 (1.68E-07)	0.00047 (0.00139)	0.0367	0.374
S14_705269	14	705269	rs210465271	Intergenic	(MIR2309)	C/T	2.1E-07 (6.76E-07)	0.00173 (0.00501)	0.0334	0.305
S14_3449027	14	3449027	rs133123595	Intergenic	—	G/A	1.14E-06 (2.79E-06)	0.00843 (0.01478)	0.0294	0.28
S11_103120099	11	103120099	rs137050272	Intergenic	(PPP2R4, IER5L)	G/A	1.69E-06 (1.28E-07)	0.01137 (0.00119)	0.0285	0.023
S14_707436	14	707436	rs436930795	Intergenic	(MIR2309)	G/A	2.17E-06 (1.43E-06)	0.01235 (0.0088)	0.0279	0.289
S14_707458	14	707458	rs470862327	Intergenic	(MIR2309)	C/T	2.17E-06 (1.43E-06)	0.01235 (0.0088)	0.0279	0.289
S14_215462	14	215462	rs133629644	Intronic	PPP1R16A	A/G	4.14E-06 (2.58E-06)	0.0192 (0.0147)	0.0264	0.262
S22_60211708	22	60211708	ss1850244253	Intergenic	—	T/G	4.73E-06 (2.13E-05)	0.02061 (0.0686)	0.0261	0.068
S3_108393170	3	108393170	rs445966307	Intronic	PTPRF	C/A	1.52E-05 (1.52E-05)	0.0564 (0.05936)	0.0233	0.018
S14_3590983	14	3590983	rs381816253	Intergenic	—	G/C	1.86E-05 (2.61E-05)	0.06276 (0.07735)	0.0228	0.381
S14_3590986	14	3590986	rs207862021	Intergenic	—	T/G	1.86E-05 (2.61E-05)	0.06276 (0.07735)	0.0228	0.381
S20_2735814	20	2735814	ss1850214424	Intergenic	—	C/A	2.29E-05 (2.79E-05)	0.0707 (0.0796)	0.0223	0.015
S14_3016578	14	3016578	rs110049376	Intergenic	(KCNK9)	G/A	2E-05 (6.39E-06)	0.06434 (0.03156)	0.0227	0.271
S17_11520990	17	11520990	ss1850176355	Intronic	EDNRA	G/T	2.63E-05 (1.68E-05)	0.07789 (0.06208)	0.022	0.018
S27_14944791	27	14944791	ss1850284750	Intergenic	(ODZ3, DCTD)	C/T	3.03E-05 (1.88E-05)	0.0832 (0.0633)	0.0217	0.021
Additive model										
S14_1092930	14	1092930	ss1850142751	Intergenic	(GRINA, Them6)	C/T	3.04E-06	0.01608	0.0271	0.37
S14_1028006	14	1028006	rs109714772	Intergenic	(GSDMD, GRINA)	G/A	5.08E-06	0.02091	0.0259	0.383
S10_26036745	10	26036745	ss1850090958	Coding^4^	TEP1	C/A	1.23E-05	0.04808	0.0238	0.016
S21_34082280	21	34082280	ss1850229276	Intronic	EDC3	G/T	3.58E-06	0.01771	0.0267	0.015
S20_38406577	20	38406577	rs466001634	Intergenic	(EGFLAM, GDNF)	G/A	2.94E-05	0.0832	0.0218	0.216
Dominant model										
S5_1569102	5	1569102	rs436392082	Intergenic	(RAB21)	A/G	3.86E-05	0.0951	0.0211	0.024
S5_1569121	5	1569121	rs382849766	Intergenic	(RAB21)	G/T	3.38E-05	0.08941	0.0214	0.024
S14_1666599	14	1666599	rs207822284	Intergenic	(LY6H)	T/C	3.98E-05	0.0951	0.0211	0.294
S14_2513747	14	2513747	rs207542860	Intronic	TRAPPC9	T/C	7.08E-06	0.03279	0.0251	0.335
S29_9009252	29	9009252	ss1850314769	Intergenic	(FZD4, PRSS23)	C/T	1.78E-05	0.06289	0.0229	0.37
S17_48121597	17	48121597	rs109745026	Intergenic	—	A/G	1.25E-05	0.05437	0.0238	0.195
S13_74353017	13	74353017	ss1850139198	Intronic	MATN4	C/A	3.83E-05	0.0951	0.0211	0.045
S2_78485721	2	78485721	ss1849982270	Intergenic	—	C/A	3.35E-05	0.08941	0.0215	0.017
S1_146045552	1	146045552	rs466469615	Intergenic	—	T/C	1.4E-05	0.05758	0.0235	0.027

^1^Near by genes are within 1Mbp of surrounding regions.

^2^Figures in brackets were obtained with the dominant model.

^3^MAF, Minor allele frequency.

^4^S10_26036745 (ss1850090958) (c.3017A>C, p.Tyr1006Ser, exon 3).

**Table 3:** Commonly significantly associated variants (minor allele frequency [MAF] ≥10%) with milk component traits.

Trait	Marker	Chr	Position	RS# or SS#	Classification	Gene	Minor/Major Allele	P-Value^1^	P-Value^1^ BH FDR	Proportion Variance Explained	MAF^2^
Associations with additive and dominant (grey highlight) models											
TD Fat%	S14_3590983	14	3590983	rs381816253	Intergenic	—	G/C	1.86E-05 (2.61E-05)	0.063 (0.077)	0.0228	0.381
	S14_3590986	14	3590986	rs207862021	Intergenic	—	T/G	1.86E-05 (2.61E-05)	0.063 (0.077)	0.0228	0.381
	S14_1092614	14	1092614	ss1850031881	Intergenic	—	A/G	5.09E-08 (1.68E-07)	0.0005 (0.001)	0.0367	0.374
	S14_714785	14	714785	ss1850173791	Intergenic	—	A/G	9.64E-09 (6.43E-09)	0.0001 (7.94E-05)	0.0406	0.315
	S14_714848	14	714848	rs207501095	Intergenic	—	G/A	6.83E-11(3.50E-10)	1.26E-06(5.19E-06)	0.0522	0.308
	S14_705269	14	705269	rs210465271	Intergenic	—	C/T	2.10E-07(6.76E-07)	0.002(0.005)	0.0334	0.305
	S14_707436	14	707436	rs436930795	Intergenic	—	G/A	2.17E-06(1.43E-06)	0.012(0.009)	0.0279	0.289
	S14_707458	14	707458	rs470862327	Intergenic	—	C/T	2.17E-06(1.43E-06)	0.012(0.009)	0.0279	0.289
	S14_1074199	14	1074199	rs210334336	Intergenic	—	T/G	2.09E-13(1.53E-11)	1.38E-08(3.77E-07)	0.0657	0.288
	S14_3449027	14	3449027	rs133123595	Intergenic	—	G/A	1.14E-06(2.79E-06)	0.008(0.015)	0.0294	0.280
	S14_392338	14	392338	rs209328075	Intergenic	—	T/C	3.72E-13(6.99E-12)	1.38E-08(2.59E-07)	0.0644	0.276
	S14_271158	14	271158	rs132685115	Intronic	TONSL	T/C	1.19E-09(3.54E-08)	1.47E-05(0.0004)	0.0455	0.2706
	S14_3016578	14	3016578	rs110049376	Intergenic	—	G/A	2.00E-05(6.39E-06)	0.064(0.032)	0.0227	0.271
	S14_215462	14	215462	ss1850067837	Intronic	PPP1R16A	A/G	4.14E-06(2.58E-06)	0.019(0.015)	0.0264	0.262
	S14_412791	14	412791	rs135576599	Intronic	ADCK5	G/A	1.23E-12(1.54E-14)	3.03E-08(1.14E-09)	0.0616	0.259
	S14_277266	14	277266	rs135581384	Intronic	TONSL	G/A	2.40E-10(2.70E-11)	3.55E-06(5E-07)	0.0493	0.257
SCC	S17_74300124	17	74300124	rs445429081	Intergenic	—	C/A	8.53E-06(1.94E-05)	0.019(0.027)	0.0247	0.153
	S16_51178060	16	51178060	ss1850171506	Intergenic	—	T/C	0.0001(2.32E-05)	0.096(0.029)	0.0185	0.127
	S18_839623	18	839623	ss1850186311	Intergenic	—	T/G	3.689E-06(7.64E-05)	0.012(0.067)	0.0267	0.123
Associations with additive model											
TD Fat%	S14_1028006	14	1028006	rs109714772	Intergenic	—	G/A	5.078E-06	0.021	0.0259	0.383
	S14_1092930	14	1092930	NA	Intergenic	—	C/T	3.038E-06	0.016	0.0271	0.370
	S20_38406577	20	38406577	rs466001634	Intergenic	—	G/A	2.938E-05	0.083	0.0218	0.216
TD Protein%	S4_114780512	4	114780512	rs384880089	Intergenic	—	C/T	5.13E-05	0.090	0.0205	0.109
SCC	S26_45210692	26	45210692	rs136238567	Intergenic	—	A/G	9.674E-05	0.083	0.019	0.340
305Fat Yield	S14_749654	14	749654	rs110892754	Intergenic	—	T/C	9.441E-07	0.070	0.0319	0.287
Associations with dominant model											
TD Fat	S14_2513747	14	2513747	rs207542860	Intronic	TRAPPC9	T/C	7.079E-06	0.033	0.025	0.335
	S14_1666599	14	1666599	rs207822284	Intergenic	—	T/C	3.977E-05	0.095	0.021	0.294
	S29_9009252	29	9009252	ss1850314769	Intergenic	—	C/T	1.782E-05	0.063	0.023	0.370
	S17_48121597	17	48121597	rs109745026	Intergenic	—	A/G	1.247E-05	0.054	0.024	0.195
TD Milk Yield	S29_9009252	29	9009252	ss1850314769	Intergenic	—	C/T	1.239E-06	0.092	0.0292	0.370
SCC	S1_47873063	1	47873063	rs210530502	Intergenic	—	A/G	9.732E-05	0.076	0.019	0.498
	S4_117466216	4	117466216	rs211180730	Intronic	CHPF2	T/C	5.837E-05	0.056	0.020	0.497
	S17_75540391	17	75540391	rs470194324	Intronic	MZT2B	G/C	0.0001309	0.090	0.018	0.198
	S4_116339811	4	116339811	rs459258791	Intronic	SSPO	C/A	4.545E-05	0.046	0.021	0.165
	S13_53375651	13	53375651	rs444484523	Intergenic	—	T/G	0.000129	0.090	0.018	0.164

^1^Values in brackets were obtained with the dominant model.

^2^MAF: Minor allele frequency.

**Table 4:** Markers^1^ showing genome wide significant associations with C20:4n6, C20:5n3, C22:5n3A, CLA:9c11t and C18:3tcc.

Marker	Chr	Position	RS# or SS#	Classification	Gene(s)	Minor/Major Allele	P-Value^2^	P-Value^2^ BH FDR	Proportion of Variance Explained	Minor Allele Frequency
C20:4n6										
Additive and Dominant models										
S19_34140957	19	34140957	rs466373116	Intronic	ADORA2B	A/G	2.86E-06 (2.87E-06)	0.012 (0.024)	0.026	0.064
S10_11170120	10	11170120	ss1850088643	Intronic	ERO1L	G/T	1.87E-07 (3.75E-06)	0.003 (0.027)	0.032	0.041
S19_39324857	19	39324857	rs466373116	Intronic	NFE2L1	G/C	5.37E-05 (1.49E-05)	0.088 (0.059)	0.019	0.035
S10_82071253	10	82071253	rs381605268	Intronic	RAD51B	A/G	2.99E-07 (4.51E-06)	0.003 (0.027)	0.031	0.017
S13_68240799	13	68240799	ss1850138245	Intronic	PPP1R16B	G/A	2.89E-06 (9.96E-08)	0.012 (0.004)	0.026	0.016
S11_99402715	11	99402715	ss1850112671	Intronic	RABEPK	C/A	1.5E-09 (3.95E-06)	0.0001 (0.027)	0.043	0.062
Additive model										
S6_95042876	6	95042876	ss1850043754	Intronic	SEPT11	A/C	2.82E-06	0.012	0.026	0.047
S4_16368721	4	16368721	ss1850009607	Intronic	ICA1	T/G	2.01E-05	0.046	0.022	0.036
S15_83503561	15	83503561	rs444340923	Downstream	OSBP	G/T	6.31E-05	0.099	0.019	0.033
S2_133356593	2	133356593	rs436496966	Upstream	LOC615263	A/G	5.07E-05	0.088	0.020	0.029
S1_158198763	1	158198763	rs478669501	Intronic	TBC1D5	C/T	1.14E-05	0.029	0.023	0.028
S23_17122196	23	17122196	rs462762139	Upstream	KLHDC3	G/T	2.63E-05	0.053	0.021	0.027
S23_25198899	23	25198899	rs109385603	Intronic	EFHC1	C/T	9.35E-06	0.028	0.023	0.023
S4_122292182	4	122292182	ss1850021400	Intronic	DNAJB6	C/A	8.05E-06	0.028	0.024	0.019
S22_453370	22	453370	rs378225721	Intronic	LANCL2	C/A	5.22E-05	0.088	0.019	0.018
S5_123328668	5	123328668	ss1850036506	Intronic	TBC1D22A	A/G	4.12E-05	0.079	0.020	0.018
S19_33423920	19	33423920	ss1850204753	Intronic	PMP22	C/A	8.39E-06	0.028	0.024	0.018
S4_117188733	4	117188733	ss1850019478	Intronic	KCNH2	C/T	6.72E-06	0.024	0.024	0.018
S3_107217401	3	107217401	ss1850001539	Intronic	C3H1orf228	G/T	1.12E-05	0.029	0.023	0.017
S5_88753240	5	88753240	ss1850030262	Intronic	ITPR2	C/A	1.05E-05	0.029	0.023	0.016
S19_33875043	19	33875043	rs385533184	Intronic	TRPV2	A/G	2.27E-05	0.049	0.021	0.016
S1_148770952	1	148770952	rs377809928	Intronic	PCBP3	T/G	4.94E-05	0.088	0.020	0.014
Dominant model										
S29_33732408	29	33732408	ss1850299837	Intronic	KCNJ1	A/C	8.62E-06	0.041	0.023	0.060
S11_10527146	11	10527146	ss1850101917	Coding	TLX2	G/C	3.03E-05	0.089	0.021	0.020
C20:5n3										
Additive and Dominant models										
S2_111911229	2	111911229	rs379338596	Intronic	TNS1	C/G	1.41E-05 (1.41E-05)	0.035 (0.070)	0.024	0.035
S14_19097860	14	19097860	ss1850146143	Intronic	PRKDC	G/T	4.25E-09 (4.94E-08)	0.0001 (0.002)	0.043	0.032
S11_39592722	11	39592722	ss1850104792	Intronic	CCDC88A	C/A	4.66E-06 (4.66E-06)	0.018 (0.039)	0.026	0.030
S3_119326797	3	119326797	rs211493744	Intronic	ATG16L1	T/C	3.06E-05 (1.91E-05)	0.057 (0.071)	0.022	0.020
S4_57933230	4	57933230	ss1850012387	Intronic	ZNF277	C/A	5.75E-11 (4.43E-06)	4.39E-06 (0.039)	0.053	0.018
S22_47121824	22	47121824	ss1850240631	Intronic	CACNA2D3	C/A	2.1E-08 (9.38E-08)	0.000 (0.002)	0.039	0.017
S24_38266364	24	38266364	rs445435952	Intronic	MYOM1	G/C	5.78E-07 (2.32E-05)	0.004 (0.081)	0.031	0.016
Additive model										
S28_43479268	28	43479268	rs470755489	Coding	ERCC6	G/C	2.49E-05	0.050	0.022	0.112
S21_55854369	21	55854369	rs437271048	Intronic	SERF2	C/T	4.62E-05	0.081	0.021	0.088
S15_7700132	15	7700132	S15_7700132	Intronic	CNTN5	G/A	4.88E-05	0.081	0.021	0.068
S20_59823962	20	59823962	rs435270372	Intronic	MYO10	C/G	6.13E-06	0.021	0.025	0.026
S15_56037691	15	56037691	rs439293424	Intronic	ACER3, CAPN5	G/T	1.88E-05	0.041	0.023	0.025
S7_61923059	7	61923059	ss1850059684	Intronic	PDGFRB	C/A	3.42E-06	0.015	0.027	0.024
S7_2124132	7	2124132	rs437404608	Intronic	ADAMTS2	A/G	3.3E-05	0.060	0.022	0.023
S29_51260084	29	51260084	ss1850305614	Intronic	LSP1	G/A	2.13E-07	0.002	0.033	0.020
S21_65175232	21	65175232	rs454925079	Intronic	EVL	G/T	3.47E-06	0.015	0.027	0.017
S10_11757086	10	11757086	ss1850088689	UTR3	RASL12	G/T	1.2E-06	0.008	0.029	0.016
S5_122892584	5	122892584	ss1850036184	Coding	TTC38	C/G	5.92E-05	0.094	0.020	0.016
Dominant model										
S3_112481449	3	112481449	rs384516162	Intronic	HPCAL4	G/C	1.55E-05	0.070	0.023	0.024
S28_473143	28	473143	ss1850289591	Intronic	NUP133	G/T	6.56E-06	0.050	0.025	0.016
C22:5n3A										
Additive and Dominant models										
S18_52444212	18	52444212	ss1850196571	Intronic	CLASRP	C/A	1.41E-06 (3.77E-06)	0.054 (0.096)	0.033	0.018
S9_106922517	9	106922517	rs466855972	Intergenic	—	T/G	4.24E-07 (2.93E-07)	0.032 (0.011)	0.036	0.140
S20_51968489	20	51968489	rs109320554	Intergenic	—	C/A	3.85E-06 (7.921E-08)	0.073 (0.006)	0.030	0.037
Additive model										
S11_50954883	11	50954883	ss1850106318	Coding	GNLY	C/G	5.67E-06	0.087	0.029	0.026
S24_17286030	24	17286030	ss1850256353	Intergenic	—	T/C	3.6E-06	0.073	0.030	0.018
CLA:9c11t										
S1_74729890	1	74729890	ss1849964962	Intergenic	—	G/A	6.01E-07 (3.05E-07)	0.046 (0.023)	0.029	0.039
C18:3tcc										
S30_5033822	X	5033822	ss1850306238	Intergenic	—	C/A	6.36E-07	0.049	0.029	0.01611

^1^Only results of markers within genes are shown for C20:4n6 and C20:5n3. Results of all markers showing genome wide significant (P value BH FDR <0.1) associations with traits listed in table are shown in Tables S5a to S5d.

^2^Values in brackets were obtained with the dominant model.

**Table 5:** Chromosomal regions (0 to 3 Mbp) harboring four or more significantly associated SNPs with the same or different fatty acid traits and termed association hot spots in this study.

Chromosomal location (size in Mbp)	Marker	Position	RS# or SS#	Minor/Major Allele	Classification	Gene(s)	P-Value	P-Value BH FDR	Proportion Variance Explained	MAF	Fatty acid
Chr 10 81299090 to 83656836 (2.36 Mbp)	S10_81299090	81299090	rs469048969	C/G	UTR3	TMEM229B	0.000679	0.08942	0.014	0.021	C13:0
	S10_81311811	81311811	rs211170312	A/G	Intronic	TMEM229B	3.65E-06	0.005572	0.025	0.037	C6:0
	S10_81311811	81311811	rs211170312	A/G	Intronic	TMEM229B	6E-05	0.064553	0.019	0.037	C8:0
	S10_81524148	81524148	rs379783190	A/C	Intergenic	—	4.91E-05	0.035386	0.019	0.017	C6:0
	S10_82071253	82071253	rs381605268	A/G	Intronic	RAD51B	2.99E-07	0.003042	0.031	0.017	**C20:4n6**
	S10_83656836	83656836	ss1850096308	G/T	Intronic	SMOC1	0.00013	0.094105	0.018	0.017	**C24:0**
Chr 11 102189029 to 103096195 (0.91 Mbp)	S11_102189029	102189029	rs472815046	G/T	Intergenic	—	2.78E-06	0.005448	0.027	0.017	**C24:0**
	S11_102513057	102513057	ss1850113339	G/T	Splicing	ODF2	7.29E-05	0.045339	0.019	0.040	**C24:0**
	S11_102912346	102912346	ss1850113453	C/A	Intronic	LRRC8A	4.84E-05	0.022963	0.019	0.032	**C13:0**
	S11_103096195	103096195	ss1850113566	C/A	Intronic	PPP2R4	2.14E-05	0.040245	0.021	0.018	C4:0
	S11_103096195	103096195	ss1850113566	C/A	Intronic	PPP2R4	1.96E-06	0.003555	0.027	0.018	C6:0
	S11_103096195	103096195	ss1850113566	C/A	Intronic	PPP2R4	0.000102	0.083004	0.019	0.018	C8:0
Chr 17 75239193 to 75857466 (0.618 Mbp)	S17_75239193	75239193	rs440208182	G/T	Downstream	MIR130B, MIR301B	3.72E-05	0.019058	0.020	0.016	**C13:0**
	S17_75451270	75451270	rs468894370	G/T	Intronic	AIFM3	0.000142	0.066741	0.018	0.023	**C24:0**
	S17_75458648	75458648	rs381895058	G/A	Intronic	LZTR1	0.000384	0.064358	0.015	0.019	**C13:0**
	S17_75636645	75636645	ss1850185522	G/A	Intronic	KLHL22	0.000432	0.089446	0.015	0.060	**C13:0**
	S17_75825603	75825603	rs207929394	T/C	Intergenic	—	1.59E-05	0.011326	0.023	0.047	**C13:0**
	S17_75857466	75857466	ss1850185673	G/T	Intronic	CDC45	2.83E-05	0.0159	0.021	0.039	**C13:0**
Chr 22 51831131 to 53880151 (2.05 Mbp)	S22_51831131	51831131	rs473131453	G/T	Intronic	SLC25A20	2.27E-06	0.003938	0.026	0.018	C6:0
	S22_51831131	51831131	rs473131453	G/T	Intronic	SLC25A20	1.82E-05	0.02778	0.022	0.018	C8:0
	S22_51965717	51965717	ss1850241844	C/A	UTR3	NCKIPSD	6.41E-06	0.00924	0.025	0.018	**C24:0**
	S22_52346974	52346974	rs110791336	T/G	Intergenic	—	0.000161	0.050409	0.017	0.066	**C13:0**
	S22_52379056	52379056	ss1850241914	G/T	Coding	CCDC51	0.000691	0.090064	0.014	0.037	**C13:0**
	S22_52380263	52380263	ss1850241922	C/T	Coding	CCDC51	0.000451	0.090988	0.015	0.022	**C13:0**
	S22_53719765	53719765	ss1850242135	G/T	Intronic	MYL3	1.48E-05	0.066302	0.027	0.015	**C23:0**
	S22_53880151	53880151	rs477695948	C/A	Coding	ALS2CL	0.000107	0.054515	0.019	0.025	**C24:0**
Chr 22 55449849 to 56791780 (1.35 Mbp)	S22_55449849	55449849	rs110927574	G/A	Intronic	GHRL	1.77E-07	0.000615	0.031996	0.0184	C6:0A
	S22_55449849	55449849	rs110927574	G/A	Intronic	GHRL	2.88E-07	0.001481	0.030908	0.0184	C8:0A
	S22_55451203	55451203	rs109410906	A/G	Intronic	GHRL	0.000111	0.075601	0.017662	0.0314	C6:0D
	S22_55494482	55494482	rs42013770	G/A	Intronic	SEC13	1.44E-06	0.002899	0.027315	0.0329	C6:0A
	S22_55494482	55494482	rs42013770	G/A	Intronic	SEC13	1.05E-05	0.020434	0.022899	0.0329	C8:0A
	S22_55554117	55554117	rs469316661	C/A	Intronic	ATP2B2	1.37E-06	0.005244	0.027425	0.0185	C4:0A
	S22_56478689	56478689	ss1850242913	G/T	Intronic	ATG7	6.25E-05	0.05658	0.018929	0.019	C6:0D
	S22_56478689	56478689	ss1850242913	G/T	Intronic	ATG7	2.83E-05	0.035383	0.02069	0.019	C8:0A
	S22_56791780	56791780	ss1850243003	A/T	Intronic	ATG7	0.000172	0.074627	0.017434	0.0694	**C24:0A**
Chr 28 41433808 to 43479268 (2.04 Mbp)	S28_41433808	41433808	rs475314514	T/C	Intronic	GLUD1	5.56E-07	0.002831	0.029443	0.0302	C4:0A
	S28_41433849	41433849	rs466502048	C/T	Intronic	GLUD1	0.000165	0.051037	0.016783	0.0155	C13:0A
	S28_41435026	41435026	ss1850132183	A/G	Intronic	GLUD1	2.95E-05	0.064795	0.020599	0.0173	**C4:0D**
	S28_41634323	41634323	ss1850295121	G/T	Intergenic	—	4.17E-05	0.03122	0.020707	0.0261	**C24:0A**
	S28_41748027	41748027	ss1850295146	C/A	Intergenic	—	5.7E-07	0.001675	0.030684	0.0165	**C24:0A**
	S28_41994199	41994199	rs42148747	T/C	Intergenic	—	0.000191	0.054817	0.016462	0.0533	**C13:0A**
	S28_42004219	42004219	rs42148747	C/A	Coding	RBP3	3.65E-05	0.01884	0.020122	0.0222	**C13:0A**
	S28_42639526	42639526	ss1850295342	C/A	Intergenic	—	7.74E-05	0.084457	0.018456	0.0168	C4:0A
	S28_43479268	43479268	rs470755489	G/C	Coding	ERCC6	2.49E-05	0.050122	0.022174	0.1121	**C20:5n3A**
Chr 29 42815829 to 45731092 (2.9 Mbp)	S29_42815829	42815829	rs447920602	G/T	Intronic	SLC22A8	0.000104	0.074691	0.017793	0.0155	C6:0D
	S29_43911453	43911453	rs132922154	C/A	Intronic	MACROD1	0.000459	0.091729	0.014533	0.0167	**C13:0A**
	S29_43949399	43949399	ss1850302054	A/G	Intronic	MACROD1	2.83E-05	0.024879	0.020684	0.0233	C6:0A
	S29_43949399	43949399	ss1850302054	A/G	Intronic	MACROD1	9.68E-05	0.082102	0.017963	0.0233	C8:0A
	S29_43950350	43950350	rs451632156	T/C	Intronic	MACROD1	4.4E-08	0.000258	0.035107	0.0161	C6:0A
	S29_43950350	43950350	rs451632156	T/C	Intronic	MACROD1	1.86E-07	0.00129	0.031889	0.0161	C8:0A
	S29_44467997	44467997	ss1850302313	C/A	Intronic	NRXN2	0.00018	0.052768	0.016598	0.0161	**C13:0A**
	S29_44684726	44684726	rs435582482	G/T	Intronic	EHD1	0.00073	0.092593	0.013517	0.0235	**C13:0D**
	S29_45566049	45566049	ss1850302687	C/G	Coding	DKFZP761E198	2.35E-06	0.002742	0.026226	0.0179	**C13:0A**
	S29_45731092	45731092	rs436614558	T/G	Coding	C29H11orf68	2.38E-05	0.014305	0.021074	0.0172	**C13:0A**

**Table 6:** Commonly significantly associated SNPs (MAF ≥ 10%) with individual fatty acid traits.

Fatty acid	Marker	Ch	Position	RS# or SS#	Classification	Gene(s)	Minor/Major Allele	P-Value^1^	P-Value^1^ BH FDR	Proportion of Variance Explained	MAF^2^
Additive and dominant models											
**C13:0**	S25_23425778	25	23425778	ss1850268922	Intergenic		G/T	0.0001(4.28E-05)	0.03736(0.018)	0.018	0.484
**C24:0**	S9_1901392	9	1901392	ss1850076897	Intronic	MYT1L	G/T	0.0002(2.74E-05)	0.075(0.040)	0.017	0.294
**C16:1**	S26_22572364	26	22572364	rs110405215	Intergenic		C/T	1.7E-08(1.23E-09)	0.001(9.4E-05)	0.037	0.225
**C22:5n3**	S9_106922517	9	106922517	rs466855972	Intergenic		T/G	4.24E-07(2.93E-07)	0.032(0.011)	0.036	0.140
C6:0	S13_54790999	13	54790999	ss1850134585	Intergenic		C/A	1.29E-05(1.12E-05)	0.014(0.022)	0.022	0.130
C8:0	S13_54790999	13	54790999	ss1850134585	Intergenic		C/A	5.85E-06(3.72E-06)	0.013(0.043)	0.024	0.130
**C13:0**	S2_121572829	2	121572829	rs43327289	Intergenic		A/G	0.0003(0.0003)	0.078(0.053)	0.015	0.110
**C13:0**	S21_65126271	21	65126271	rs209872748	Intronic	EVL	G/A	5.06E-05(0.0006)	0.024(0.081)	0.019	0.110
**C23:0**	S2_21504102	2	21504102	ss1849977641	Intergenic		C/A	1.9E-05(2.56E-05)	0.096(0.0723)	0.027	0.106
C4:0	S9_104910843	9	104910843	rs379509819	Intergenic		C/T	3.59E-05(3.8E-06)	0.053(0.017)	0.02	0.104
Additive model											
**C23:0**	S19_60864657	19	60864657	rs136101412	Intergenic		C/G	1.33E-05	0.09199	0.028	0.488
C4:0	S28_39747506	28	39747506	rs210570524	Intergenic		C/G	1.44E-05	0.02929	0.022	0.484
C4:0	S21_19933271	21	19933271	rs42850142	Intronic	LOC512150	T/C	0.000119	0.0999	0.018	0.463
C8:0	S23_15260292	23	15260292	rs453839711	Intergenic		A/C	1.82E-05	0.02778	0.022	0.429
**C24:0**	S3_125969441	3	125969441	rs439184439	Intergenic		G/A	8.19E-05	0.04775	0.019	0.394
C6:0	S21_65164887	21	65164887	rs432423874	Intronic	EVL	G/A	0.000191	0.09392	0.016	0.365
**C20:5n3**	S24_40818403	24	40818403	rs109764724	Intergenic		C/T	6.74E-06	0.02144	0.025	0.357
C4:0	S23_44081116	23	44081116	rs42030314	Intergenic		C/A	8.52E-05	0.088	0.018	0.323
C4:0	S15_2204384	15	2204384	rs133014695	Intergenic		A/G	3.68E-05	0.05308	0.02	0.320
**C24:0**	S8_1667147	8	1667147	rs109630798	Intergenic		G/A	0.000119	0.05878	0.018	0.280
**C24:0**	S15_84435339	15	84435339	rs110379736	Intergenic		A/G	8.15E-05	0.04775	0.019	0.265
**C24:0**	S28_44246992	28	44246992	rs42155039	Intronic	MARCH8	T/C	8.18E-05	0.04775	0.019	0.226
C4:0	S18_34969902	18	34969902	rs383910840	Intronic	CDH1	A/G	7.33E-05	0.08446	0.019	0.212
C8:0	S6_84481246	6	84481246	ss1850043060	Intergenic		C/T	7.76E-05	0.07165	0.018	0.212
**C11:0**	S6_84481246	6	84481246	ss1850043060	Intergenic		C/T	2.33E-06	0.0889	0.026	0.211
**C13:0**	S11_6782323	11	6782323	rs207538712	Intergenic		C/T	0.000384	0.08464	0.015	0.192
**C24:0**	S18_26865059	18	26865059	rs209759354	Intergenic		G/A	0.000229	0.08772	0.017	0.191
**C24:0**	S11_69400708	11	69400708	ss1850107740	Intronic	ANTXR1	C/T	0.000278	0.09614	0.016	0.186
C4:0	S10_104486629	10	104486629	rs109791124	Intergenic		G/A	6.59E-06	0.01937	0.024	0.185
C4:0	S28_24883940	28	24883940	rs208750352	Intronic	HK1	T/C	3.65E-05	0.05308	0.02	0.179
C4:0	S28_24883941	28	24883941	rs210345641	Intronic	HK1	T/C	3.65E-05	0.05308	0.02	0.179
**C13:0**	S24_60265567	24	60265567	ss1850262310	Intergenic		T/C	0.000396	0.08542	0.015	0.173
C6:0	S18_5423381	18	5423381	rs41856683	Intergenic		A/G	1.03E-05	0.01212	0.023	0.158
C8:0	S18_5423381	18	5423381	rs41856683	Intergenic		A/G	4.24E-06	0.01045	0.025	0.158
**C24:0**	S6_106786751	6	106786751	rs133693494	Intergenic		C/T	0.000117	0.05846	0.018	0.151
**C13:0**	S15_64028144	15	64028144	rs41776609	Intronic	CD59	T/C	7.75E-05	0.03149	0.018	0.142
**C13:0**	S11_94106253	11	94106253	rs135845527	Intergenic		G/C	0.000323	0.07741	0.015	0.133
**C24:0**	S3_25023007	3	25023007	rs135236823	Intergenic		G/C	0.000132	0.063	0.018	0.132
C6:0	S8_88522633	8	88522633	rs463987848	Intronic	BICD2	C/T	0.000177	0.0907	0.017	0.131
C4:0	S4_119339436	4	119339436	rs378444093	Intergenic		T/C	0.000116	0.09929	0.018	0.130
C4:0	S4_119339451	4	119339451	rs381758710	Intergenic		A/G	0.000116	0.09929	0.018	0.130
**C13:0**	S13_44358829	13	44358829	ss1850314106	Intergenic		C/G	0.000491	0.09547	0.014	0.128
C4:0	S9_106369166	9	106369166	ss1850086265	Intergenic		A/G	0.000112	0.09853	0.018	0.116
C4:0	S9_106369170	9	106369170	rs470906002	Intergenic		A/G	0.000112	0.09853	0.018	0.116
**C13:0**	S4_4330240	4	4330240	rs207853593	Intergenic		T/C	0.000215	0.06039	0.016	0.116
**C20:5n3**	S28_43479268	28	43479268	rs470755489	Coding	ERCC6	G/C	2.49E-05	0.05012	0.022	0.112
C6:0	S21_31073148	21	31073148	rs207776812	Intronic	PSMA4	T/A	0.000205	0.09734	0.016	0.103
C8:0	S21_31073148	21	31073148	rs207776812	Intronic	PSMA4	T/A	7.82E-05	0.07165	0.018	0.103
Dominant model											
**C13:0**	S21_26707088	21	26707088	rs110179128	Intergenic		T/C	0.000395	0.06482	0.015	0.482
**C23:0**	S8_58122774	8	58122774	rs135761549	Intergenic		C/T	2.7E-05	0.07352	0.026	0.397
C6:0	S13_83809385	13	83809385	rs208127058	Intergenic		G/A	9.73E-05	0.07238	0.018	0.360
**SFA**	S17_74491357	17	74491357	rs41855732	Intergenic		G/C	1.38E-06	0.05285	0.027	0.328
**C18:1n9c**	S17_74491357	17	74491357	rs41855732	Intergenic		G/C	3.3E-06	0.08392	0.025	0.328
**MUFA**	S17_74491357	17	74491357	rs41855732	Intergenic		G/C	2.39E-06	0.05623	0.026	0.328
**C23:0**	S2_94271418	2	94271418	rs481169913	Intergenic		C/A	5.37E-06	0.03054	0.03	0.324
**SFA**	S16_48622152	16	48622152	rs465358414	Intergenic		G/C	1.03E-05	0.09875	0.023	0.270
**SFA**	S14_277266	14	277266	rs135581384	Intronic	TONSL	G/A	3.48E-06	0.07814	0.025	0.260
**C18:1n9c**	S14_277266	14	277266	rs135581384	Intronic	TONSL	G/A	1.53E-06	0.05824	0.027	0.260
**C24:0**	S10_20831159	10	20831159	rs211405588	Intergenic		T/C	0.000146	0.09791	0.018	0.243
**C4:0**	S25_11286202	25	11286202	rs109779107	Intergenic		G/C	2.22E-05	0.0584	0.021	0.226
**C13:0**	S29_8375318	29	8375318	rs208360098	Intergenic		C/T	0.000692	0.09006	0.014	0.219
**C13:0**	S29_8375314	29	8375314	rs211269917	Intergenic		T/C	0.000833	0.09972	0.013	0.216
**C13:0**	S1_130615136	1	130615136	rs208659424	Intronic	CLSTN2	T/C	0.000196	0.04411	0.016	0.209
**C13:0**	S27_39615226	27	39615226	rs135921072	Intergenic		C/T	0.000202	0.04533	0.016	0.202
**C13:0**	S8_97716573	8	97716573	rs135846930	Intergenic		T/C	0.000547	0.07852	0.014	0.199
**C13:0**	S27_39607188	27	39607188	rs207920200	Intergenic		C/T	0.00052	0.07685	0.014	0.191
**C23:0**	S10_100984214	10	100984214	rs110395591	Intergenic		C/T	2.34E-05	0.07249	0.026	0.174
**C20:4n6**	S27_16628049	27	16628049	rs42116637	Intergenic		A/C	5.03E-06	0.02745	0.025	0.169
**C24:0**	S1_4948474	1	4948474	rs137452899	Intergenic		A/G	7.62E-05	0.07742	0.019	0.164
**C13:0**	S25_38252119	25	38252119	rs108953935	Upstream	PILRA	A/G	0.000776	0.09531	0.013	0.156
**C23:0**	S10_100494903	10	100494903	rs380814997	Intergenic		T/C	2.43E-05	0.07249	0.026	0.151
**C13:0**	S1_130624357	1	130624357	rs210558202	Intronic	CLSTN2	T/C	0.000767	0.09461	0.013	0.143
**C13:0**	S26_48673391	26	48673391	rs133171238	Intergenic		A/G	7.55E-05	0.02452	0.019	0.135
**C24:0**	S8_14848948	8	14848948	rs110198610	Intergenic		G/C	0.000133	0.09462	0.018	0.133
**C24:0**	S8_14848943	8	14848943	rs110923026	Intergenic		T/C	9.01E-05	0.07814	0.019	0.132
**C13:0**	S2_121574985	2	121574985	rs109350928	Intergenic		A/G	0.000322	0.05826	0.015	0.112
**C23:0**	S17_47372617	17	47372617	ss1850179088	Intergenic		C/T	5E-05	0.09826	0.024	0.11
**C13:0**	S2_125566351	2	125566351	rs110334370	Intergenic		A/G	0.000317	0.05775	0.015	0.11
**C4:0**	S9_104908398	9	104908398	rs382936674	Intergenic		G/A	7.4E-06	0.02826	0.024	0.108
C6:0	S12_84413041	12	84413041	rs136905662	Coding	F7	T/G	0.000105	0.07469	0.018	0.104
**C4:0**	S9_104910742	9	104910742	rs467296387	Intergenic		T/C	1.31E-05	0.03991	0.022	0.101

^1^Values in brackets were obtained with the dominant model.

^2^MAF: Minor allele frequency.
