# Supplementary material for: High density genome wide genotyping-by-sequencing and association identifies common and low frequency SNPs, and novel candidate genes influencing cow milk traits
Source: Sci Rep. 2016 Aug 10;6:31109. doi: 10.1038/srep31109 (PMC4979022; doi:10.1038/srep31109)
Supplement: Supplementary Information [file srep31109-s1.pdf]

High density genome wide genotyping-by-sequencing and association identifies common and low frequency SNPs, and novel candidate genes influencing cow milk traits

Eveline M. Ibeagha-Awemu[1*], Sunday O. Peters[2], Kingsley A. Akwanji[3], Ikhide G. Imumorin[4], Xin Zhao[3*]

[1]Agriculture and Agri-Food Canada, Sherbrooke Research and Development Centre, 2000 Rue College, Sherbrooke, QC, J1M 0C8 Canada;

[2]Department of Animal Science, Berry College, Mount Berry, GA 30149 USA;

[3]Department of Animal Science, McGill University, 21,111 Lakeshore Road, Ste-Anne-De-Bellevue, QC, H9S 3V9 Canada;

[4]Animal Genetics & Genomics Lab., International Programs, College of Agriculture & Life Sciences, Cornell University, Ithaca, NY 14853 USA

Correspondence

Dr. Eveline Ibeagha-Awemu

Agriculture and Agri-Food Canada

Sherbrooke Research and Development Center, Sherbrooke, QC, Canada

Eveline.ibeagha-awemu@agr.gc.ca

Dr Xin Zhao

Department of Animal Science,

McGill University, Ste-Anne-De-Bellevue, QC, Canada

Xin.zhao@mcgill.ca

Supplemetary figures

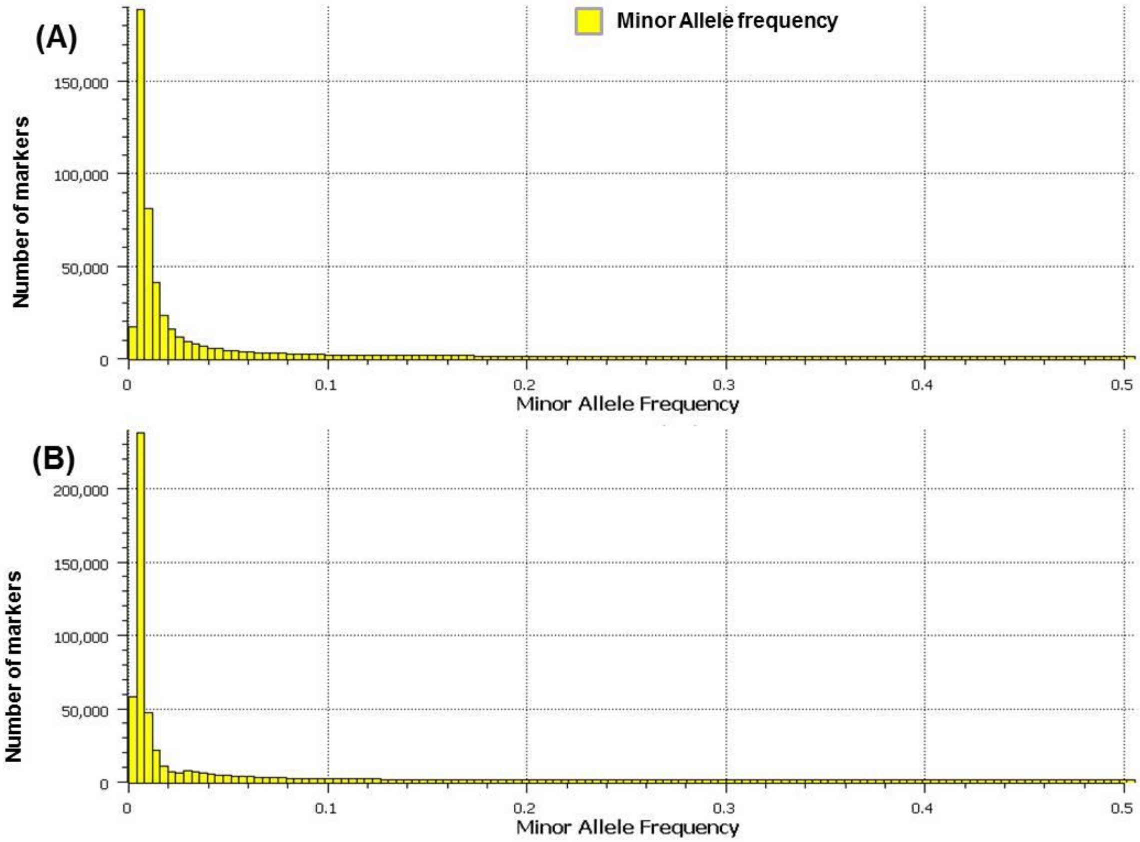

Figure S1: Minor allele frequencies before (A) and after (B) genotype imputation

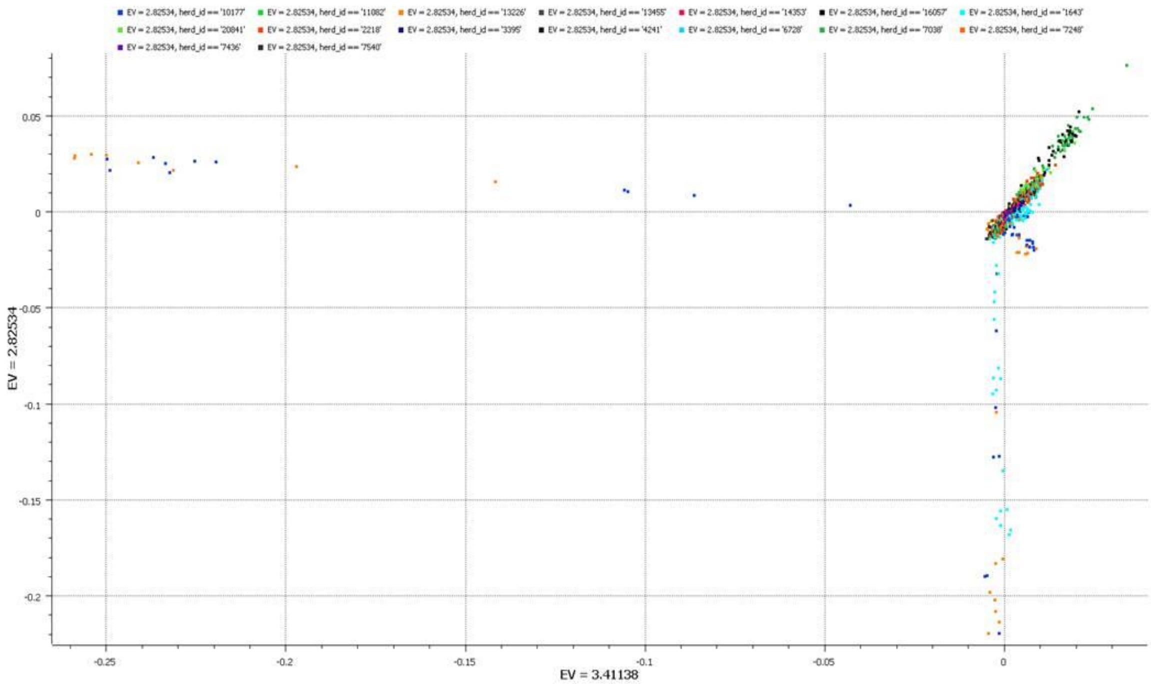

Figure S2: A scatterplot of the first two principal components based on the whole unfiltered genotype dataset. The first PC is on the X-axis, the second component is on the Y-axis and the plot is colored by herd (16 herds in total). Three herds appear to be inconsistent with majority of the data with regards to the first two PCs.

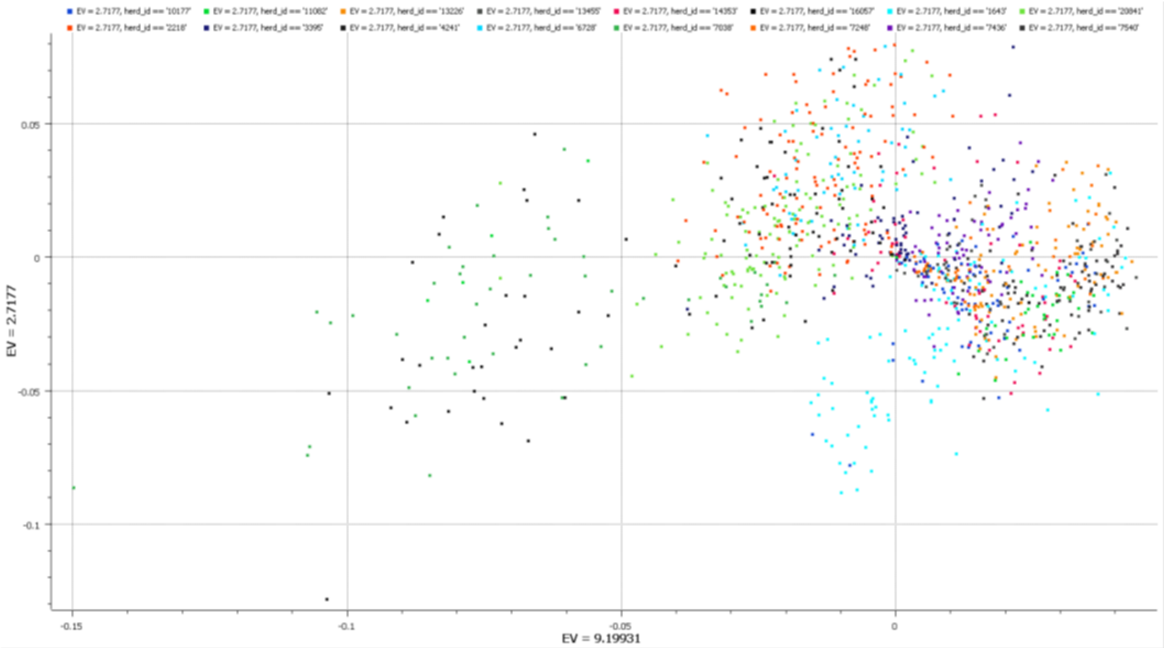

Figure S3: A scatterplot of the first two principal components (PC) after restricting the markers to moderately high call rates (≥ 80%) and minor allele frequencies (≥ 1%). The first PC is on the X-axis, the second component is on the Y-axis and the plot is colored by herd (16 herds in total). The three herds that appeared to be outliers in the previous plot (Figure S2) are now consistent with the majority of the data.

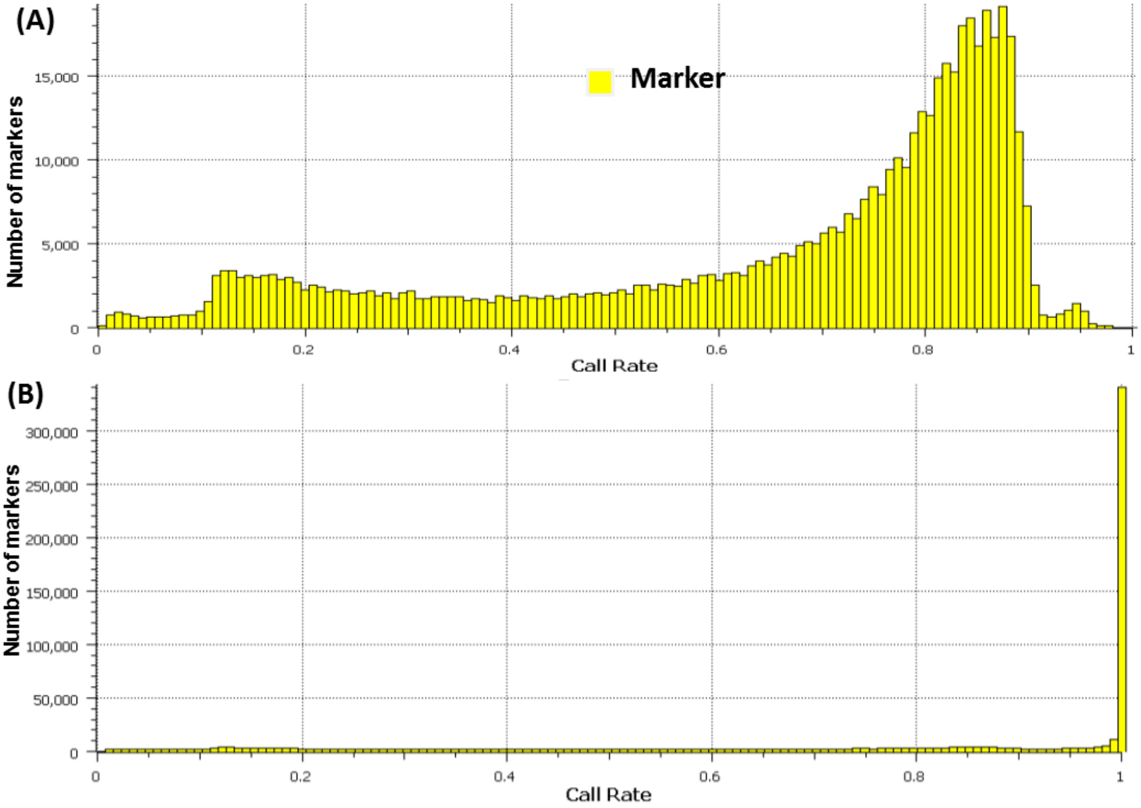

Figure S4: Marker call rates before (A) and after (B) genotype imputation.

Supplementary tables and titles

Table S1**:** Reads and mapping statistics

FastQ file	Barcodes found in lane	Total # of reads per lane	Total number of good barcoded reads	Resulting # of Tags
C0DBVACXX_8_fastq.gz	96	203828046	181673602	5481845
C0TCDACXX_3_fastq.gz	96	293283529	263270239	6784427
C0TCDACXX_5_fastq.gz	96	276686206	214141708	8601994
C0TCDACXX_6_fastq.gz	96	294963258	101156680	5090969
C0TCDACXX_7_fastq.gz	96	273856276	252902231	6192788
C0TCDACXX_8_fastq.gz	96	295162436	218547395	6167941
C12A1ACXX_1_fastq.gz	96	201766712	178608568	5243562
C12A1ACXX_2_fastq.gz	96	289041244	167367096	6607205
C12A1ACXX_3_fastq.gz	96	241868755	214603016	6796276
C12A1ACXX_5_fastq.gz	96	247859392	220413999	6959808
C12A1ACXX_6_fastq.gz	96	250344775	223724434	7006117
C12A1ACXX_7_fastq.gz	96	228480895	202173353	6206376
C14VRACXX_8_fastq.gz	96	231740161	206123597	6355048
D0E1YACXX_7_fastq.gz	96	166518190	66743149	2990986
D17NUACXX_3_fastq.gz	96	199103718	176718010	6182956
Total		3,694,503,593	2,888,167,077	92,668,298
Total number of merged tags				14,635,202
% uniquely aligned tags				79.6
% multiple aligned tags				10.5
% not aligned tags				9.9

Table S2: Distribution of identified markers by chromosome, variant classification and coding variant classes

**BTA**	**Markers**
1	23383
2	22750
3	23860
4	21214
5	22403
6	17139
7	21511
8	18111
9	14986
10	18588
11	25093
12	14240
13	21182
14	15078
15	16045
16	17108
17	16381
18	20004
19	21549
20	13563
21	16472
22	15674
23	13123
24	13409
25	17652
26	11557
27	9650
28	9965
29	14626
X	9471
**Total**	**515787**

**Variant classification**	**Number**
Intergenic	353252
Downstream	3921
Upstream	4130
Intronic	127988
Non coding exonic	78
3'Untranslated region	2774
5'Untranslated region	807
Splicing	858
Coding	17699
***Total**	**511507**

**Coding variant classes**	**Number**
Unknown	2027
Synonymous	3306
Non-synonymous	12264
Stop loss	34
Stop gain	59
Initiation codon	9
Splicing	858
**Total**	**18557**

*A total of 4280 variants on 367 invalid transcripts could not be classified

Table S3a to b: Single nucleotide polymorphisms identified in this study: SNPs with RS (a) or SS numbers (b)
(xlsx) (Included as supplementary data set)

Table S4a to e: Significant genome wide associations between studied markers and test day fat percentage (TFP) (a), test day protein percentage (TPP) (b), milk somatic cell counts (SCC) (c), test day milk yield (kg) and actual 305day fat yield (kg) (d), milk urea nitrogen (MUN) (mg/dl) and lactose (%) (e)
(xlsx) (Included as supplementary data set)

Table S5a to d: Significant genome wide associations between studied markers and C20:5n3 (eicosapentaenoic acid, EPA) (a), C20:4n6 (arachidonic acid, AA) (b), C22:5n3 (docosapentaenoic acid, DPA) (c), CLA:9c11t and C18:3tcc (d)
(xlsx) (Included as supplementary data set)

Table S6a to b: Significant genome wide associations between studied markers and palmitoleic acid (C16:1) and oleic acid (C18:1n9c) (a), and total monounsaturated fatty acid (MUFA) (b)
(xlsx) (Included as supplementary data set)

Table S7a to h: Significant genome wide associations between studied markers and butyric acid (C4:0) (a), caproic acid (C6:0) (b), caprylic acid (C8:0) (c), undecanoic acid (C11:0) , myristic acid (C14:0), pentadecylic acid (C15:0) and margaric acid (C17:0) (d), tridecylic acid (C13:0) (e), tricosanoic acid (C23:0) (f), lignoceric acid (C24:0) (g) and total saturated fatty acid (SFA) (h)
(xlsx) (Included as supplementary data set)

Table S8: Several SNPs on the same gene significantly associated with the same or different fatty acids

Gene(s)	Marker	Chr	Position	RS# or SS#	Classification	Minor Allele	Major Allele	P-Value	P-Value BH FDR	Proportion of Variance Explained	Minor Allele D Frequency	Fatty acid
GRB10	S4_5334758	4	5334758	ss1850008192	Intronic	G	C	1.1821E-06	0.00159	0.0278	0.0166	C13:0
	S4_5334761	4	5334761	ss1850008194	Intronic	C	G	4.81042E-05	0.02296	0.0195	0.0166	C13:0
	S4_5349547	4	5349547	ss1850008216	Intronic	G	T	1.35204E-05	0.01484	0.0233	0.0161	C24:0
THSD7A	S4_20307923	4	20307923	rs462192019	Intronic	G	A	3.97797E-05	0.03131	0.0199	0.0393	C6:0
	S4_20307923	4	20307923	rs462192019	Intronic	G	A	3.87155E-05	0.07778	0.02	0.0393	C8:0
CAMK2B	S4_79346368	4	79346368	ss1850014993	Intronic	A	C	2.68905E-05	0.01555	0.0208	0.018	C13:0
	S4_79360395	4	79360395	rs469668684	Intronic	G	T	1.62823E-07	0.00059	0.0322	0.0179	C6:0
	S4_79360395	4	79360395	rs469668684	Intronic	G	T	2.1646E-06	0.00661	0.0264	0.0179	C8:0
LRRC4, SND1	S4_94911118	4	94911118	rs207689820	Intronic	T	C	1.08388E-05	0.08276	0.0282	0.0501	C23:0
	S4_94911147	4	94911147	rs209422045	Intronic	T	C	2.65467E-06	0.02534	0.0321	0.0501	C23: 0
DPP6	S4_120202766	4	120202766	rs209121473	Intronic	C	T	8.88954E-05	0.0336	0.0182	0.0202	C13:0
	S4_120299015	4	120299015	ss1850020368	Intronic	C	A	5.28553E-05	0.03669	0.0202	0.031	C24:0
PAXIP1	S4_120527040	4	120527040	ss1850020600	Coding	G	T	0.000126682	0.08061	0.0174	0.0179	C6:0
	S4_120539350	4	120539350	ss1850020621	Intronic	C	A	6.02105E-06	0.00836	0.0241	0.0187	C6:0
	S4_120539350	4	120539350	ss1850020621	Intronic	C	A	3.60871E-05	0.04374	0.0201	0.0187	C8:0
VIPR2	S4_123299193	4	123299193	ss1850022099	Intronic	C	T	4.48026E-05	0.03421	0.0197	0.0162	C6:0
	S4_123299193	4	123299193	ss1850022099	Intronic	C	T	8.79428E-05	0.07545	0.0182	0.0162	C8:0
TFCP2	S5_31462407	5	31462407	rs462777283	Intronic	G	T	2.40726E-05	0.02215	0.021	0.0161	C6:0
	S5_31462407	5	31462407	rs462777283	Intronic	G	T	5.56188E-05	0.06067	0.0192	0.0161	C8:0
NR1H4	S5_69070798	5	69070798	rs378694894	Intronic	C	A	0.000421056	0.08808	0.0147	0.0236	C13:0
	S5_69076535	5	69076535	ss1850028073	Intronic	G	T	0.000296077	0.05582	0.0155	0.0161	C13:0
TTC38	S5_122892584	5	122892584	ss1850036184	Coding	C	G	5.9224E-05	0.09391	0.0201	0.0157	C20:5n3
	S5_122902343	5	122902343	ss1850313753	Intronic	C	A	1.9566E-05	0.0957	0.0266	0.0162	C23:
TBC1D22A	S5_123328534	5	123328534	ss1850036502	Intronic	A	C	0.000766182	0.09461	0.0134	0.0342	C13:0
Intronic	S5_123328668	5	123328668	ss1850036506	+	A	1			0.02	0.0179	C20:4n6
TBC1D22A	S5_123401529	5	123401529	rs465458272	Intronic	T	C	4.50285E-05	0.02204	0.0197	0.0665	C13:0

ACOX3	S6_121138698	6	121138698	ss1850048597	Intronic	G	T	6.19873E-09	0.00012	0.0395	0.0197	C4:0
	S6_121138698	6	121138698	ss1850048597	Intronic	G	T	1.74458E-08	0.00015	0.0372	0.0197	C6:0
	S6_121138698	6	121138698	ss1850048597	Intronic	G	T	2.08922E-06	0.00661	0.0265	0.0197	C8:0
HBEGF	S7_50817144	7	50817144	ss1850058473	Intronic	C	A	7.83091E-06	0.01013	0.0235	0.0409	C6:0
	S7_50817144	7	50817144	ss1850058473	Intronic	C	A	0.000103272	0.083	0.0178	0.0409	C8:0
GFRA2	S8_71948968	8	71948968	ss1850070776	Intronic	G	T	7.83603E-05	0.06365	0.0184	0.0162	C6:0
	S8_71996796	8	71996796	ss1850070800	Intronic	G	T	0.00021615	0.06045	0.0162	0.0226	C13:0
PALM2	S8_104441473	8	104441473	ss1850074571	Intronic	A	C	1.10115E-07	0.00044	0.0331	0.0192	C6:0
	S8_104441473	8	104441473	ss1850074571	Intronic	A	C	3.15946E-06	0.00928	0.0256	0.0192	C8:0
COLEC11	S8_116299522	8	116299522	ss1850076308	Intronic	C	A	4.00488E-06	0.006	0.025	0.0173	C6:0
	S8_116299522	8	116299522	ss1850076308	Intronic	C	A	1.47241E-05	0.02498	0.0221	0.0173	C8:0
TMEM229B	S10_81299090	10	81299090	rs469048969	UTR3	C	G	0.000679472	0.08942	0.0137	0.0207	C13:0
	S10_81311811	10	81311811	rs211170312	Intronic	A	G	3.64891E-06	0.00557	0.0252	0.0372	C6:0
	S10_81311811	10	81311811	rs211170312	Intronic	A	G	6.0026E-05	0.06455	0.019	0.0372	C8:0
NRXN3	S10_92846704	10	92846704	rs379631598	Intronic	C	T	0.000503307	0.09754	0.0143	0.0183	C13:0
	S10_93188395	10	93188395	rs43649533	Intronic	C	G	1.26979E-06	0.0889	0.0277	0.0185	C11:0
NPAS2	S11_6130664	11	6130664	rs211557881	Intronic	A	G	4.41443E-08	1E-04	0.0351	0.0165	C13:0
	S11_6130715	11	6130715	rs208606161	Intronic	C	G	4.43636E-08	1E-04	0.0351	0.0165	C13:0
	S11_6131052	11	6131052	rs208416548	Intronic	T	C	0.000447091	0.09099	0.0146	0.0151	C13:0
	S11_6131087	11	6131087	rs381819658	Intronic	C	T	0.000447091	0.09099	0.0146	0.0151	C13:0
	S11_6131088	11	6131088	rs385362063	Intronic	A	G	0.000447091	0.09099	0.0146	0.0151	C13:0
PPP2R4	S11_103096195	11	103096195	ss1850113566	Intronic	C	A	2.14098E-05	0.04024	0.0213	0.0184	C4:0
	S11_103096195	11	103096195	ss1850113566	Intronic	C	A	1.95523E-06	0.00355	0.0266	0.0184	C6:0
	S11_103096195	11	103096195	ss1850113566	Intronic	C	A	0.000102077	0.083	0.0178	0.0184	C8:0
NEBL	S13_21485963	13	21485963	ss1850129455	Intronic	G	T	0.000103112	0.06151	0.0178	0.0167	C6:0
	S13_21485963	13	21485963	ss1850129455	Intronic	G	T	7.65325E-05	0.07165	0.0185	0.0167	C8:0
RSU1	S13_30840582	13	30840582	rs451505068	Downstream	G	T	1.25425E-05	0.01408	0.0225	0.019	C6:0
	S13_30840582	13	30840582	rs451505068	Downstream	G	T	0.000102753	0.083	0.0178	0.019	C8:0
KCNB1	S13_78331772	13	78331772	ss1850140320	Coding	G	T	0.000143558	0.07931	0.0171	0.0178	C6:0
	S13_78331772	13	78331772	ss1850140320	Coding	G	T	1.03612E-05	0.02043	0.0229	0.0178	C8:0

PLXNA2	S16_73395602	16	73395602	ss1850174098	Intronic	G	T	1.4634E-05	0.00916	0.0222	0.0182	C13:0
	S16_73448076	16	73448076	rs476345002	Coding	C	A	0.000139666	0.04629	0.0172	0.0272	C13:0
GGT6	S19_24964715	19	24964715	rs458879791	Intronic	G	T	1.20388E-10	9.2E-06	0.0482	0.0154	C4:0
	S19_24964715	19	24964715	rs458879791	Intronic	G	T	8.57981E-15	6.6E-10	0.0693	0.0154	C6:0
	S19_24964715	19	24964715	rs458879791	Intronic	G	T	5.03359E-12	3.8E-07	0.0553	0.0154	C8:0
NBR1	S19_44174585	19	44174585	ss1850207539	Coding	C	A	0.000199693	0.09551	0.0164	0.0161	C6:0
	S19_44174585	19	44174585	ss1850207539	Coding	C	A	0.000134961	0.09909	0.0172	0.0161	C8:0
SOST	S19_44670572	19	44670572	ss1850207671	Intronic	C	G	9.8663E-05	0.03587	0.0179	0.0155	C13:0
	S19_44672003	19	44672003	ss1850207675	Upstream	G	C	0.000124267	0.07188	0.0174	0.0207	C6:0
	S19_44672003	19	44672003	ss1850207675	Upstream	G	C	8.3249E-05	0.07276	0.0183	0.0207	C8:0
WIPI1	S19_63354466	19	63354466	rs481559924	Intronic	T	G	2.10232E-07	0.00401	0.039	0.0633	C23:
	S19_63483972	19	63483972	ss1850213316	Intronic	C	A	1.68323E-05	0.01149	0.0218	0.0212	C13:0
KLHL25	S21_16203091	21	16203091	ss1850225567	Intronic	T	A	2.19044E-08	0.00013	0.0383	0.0226	C24:0
	S21_16203094	21	16203094	ss1850225569	Intronic	T	C	3.94964E-05	0.05026	0.0208	0.0396	C24:0
EVL	S21_65116987	21	65116987	rs381368835	Upstream	T	G	0.000542962	0.07852	0.0142	0.0242	C13:0
	S21_65116987	21	65116987	rs381368835	Upstream	T	G	4.62117E-05	0.09826	0.0242	0.0276	C23:0
	S21_65116987	21	65116987	rs381368835	Upstream	T	G	0.000146176	0.09791	0.0178	0.0239	C24:0
	S21_65126271	21	65126271	rs209872748	Intronic	G	A	5.06007E-05	0.02352	0.0194	0.1098	C13:0
	S21_65164883	21	65164883	rs467244058	Intronic	C	G	0.000129932	0.07404	0.0173	0.0208	C6:0
	S21_65164887	21	65164887	rs432423874	Intronic	G	A	0.000190655	0.09392	0.0165	0.3652	C6:0
	S21_65175232	21	65175232	rs454925079	Intronic	G	T	3.4715E-06	0.01455	0.0268	0.017	C20:5n3
SLC25A20	S22_51831131	22	51831131	rs473131453	Intronic	G	T	2.26909E-06	0.00394	0.0263	0.0179	C6:0
	S22_51831131	22	51831131	rs473131453	Intronic	G	T	1.81961E-05	0.02778	0.0217	0.0179	C8:0
CCDC51	S22_52379056	22	52379056	ss1850241914	Coding	G	T	0.000690756	0.09006	0.0136	0.0367	C13:0
	S22_52380263	22	52380263	ss1850241922	Coding	C	T	0.000451292	0.09099	0.0146	0.0221	C13:0
GHRL	S22_55449849	22	55449849	rs110927574	Intronic	G	A	1.7721E-07	0.00062	0.032	0.0184	C6:0
	S22_55449849	22	55449849	rs110927574	Intronic	G	A	2.88444E-07	0.00148	0.0309	0.0184	C8:0
	S22_55451203	22	55451203	rs109410906	Intronic	A	G	0.000110894	0.0756	0.0177	0.0314	C6:0
ATG7	S22_56478689	22	56478689	ss1850242913	Intronic	G	T	6.25373E-05	0.05658	0.0189	0.019	C6:0
	S22_56478689	22	56478689	ss1850242913	Intronic	G	T	2.82671E-05	0.03538	0.0207	0.019	C8:0

	S22_56791780	22	56791780	ss1850243003	Intronic	A	T	0.000172017	0.07463	0.0174	0.0694	C24:0
CCDC51	S22_52379056	22	52379056	ss1850241914	Coding	G	T	0.000690756	0.09006	0.0136	0.0367	C13:0
	S22_52380263	22	52380263	ss1850241922	Coding	C	T	0.000451292	0.09099	0.0146	0.0221	C13:0
CFB	S23_26203835	23	26203835	ss1850249533	Coding	G	A	1.64889E-05	0.01749	0.0219	0.0186	C6:0
	S23_26203835	23	26203835	ss1850249533	Coding	G	A	2.11172E-05	0.06248	0.0213	0.0186	C8:0
PHACTR1	S23_43066407	23	43066407	ss1850251288	Intronic	C	A	4.08621E-08	0.00026	0.0353	0.0204	C6:0
	S23_43066407	23	43066407	ss1850251288	Intronic	C	A	1.79758E-08	0.00034	0.0371	0.0204	C8:0
	S23_43275147	23	43275147	rs210642353	Intronic	C	G	0.000184502	0.07615	0.0173	0.0199	C24:0
FARS2	S23_49090745	23	49090745	ss1850252486	Intronic	G	A	0.000100247	0.05243	0.0187	0.0211	C24:0
	S23_49259135	23	49259135	ss1850252556	Intronic	G	T	0.000168712	0.05155	0.0167	0.0202	C13:0
GMDS	S23_51589318	23	51589318	ss1850253428	Intronic	G	T	0.000108432	0.03066	0.0177	0.0166	C13:0
	S23_51592262	23	51592262	ss1850253438	Intronic	C	A	6.72332E-15	5.1E-10	0.0728	0.0155	C24:0
ATP9B	S24_619353	24	619353	ss1850254132	Intronic	C	A	2.16118E-05	0.07249	0.0263	0.0144	C23:0
	S24_749043	24	749043	rs381059800	Intronic	T	C	3.86631E-05	0.01968	0.02	0.0209	C13:0
BCL2	S24_63724092	24	63724092	rs447857210	Intronic	C	A	2.10546E-08	0.00032	0.0367	0.0161	C4:0
	S24_63724092	24	63724092	rs447857210	Intronic	C	A	1.8963E-11	7.2E-07	0.0523	0.0161	C6:0
	S24_63724092	24	63724092	rs447857210	Intronic	C	A	4.35944E-10	1.7E-05	0.0454	0.0161	C8:0
NPRL3	S25_612572	25	612572	ss1850263261	Coding	C	A	3.49786E-05	0.02782	0.0202	0.0287	C6:0
	S25_615463	25	615463	ss1850263264	Intronic	C	A	2.92928E-05	0.02485	0.0215	0.0181	C24:0
RPUSD1	S25_1124112	25	1124112	rs437044361	UTR3	C	A	3.09893E-05	0.01678	0.0205	0.0173	C13:0
	S25_1125561	25	1125561	rs210217478	Intronic	T	C	2.10464E-05	0.07249	0.0264	0.0573	C23:0
RBFOX1	S25_7154373	25	7154373	ss1850266283	Intronic	C	A	0.000137051	0.077	0.0172	0.0447	C6:0
	S25_7154373	25	7154373	ss1850266283	Intronic	C	A	0.000137301	0.09984	0.0172	0.0447	C8:0
MAD1L1	S25_42883541	25	42883541	ss1850273631	Coding	G	A	0.000459322	0.07145	0.0145	0.0371	C13:0
	S25_42889432	25	42889432	ss1850273649	Intronic	T	G	8.59897E-05	0.04793	0.019	0.028	C24:0
	S25_42945228	25	42945228	ss1850273705	Intronic	C	A	0.000279613	0.05411	0.0156	0.0191	C13:0
	S25_42961862	25	42961862	ss1850314685	Intronic	A	C	9.45535E-06	0.01128	0.0231	0.0241	C6:0
ADAM12	S26_46502453	26	46502453	ss1850280485	Intronic	T	C	0.000140838	0.04629	0.0171	0.0344	C13:0
	S26_46820798	26	46820798	rs379603734	Intronic	C	T	2.78693E-05	0.04729	0.0207	0.0375	C4:0
	S26_46820843	26	46820843	rs385414454	Intronic	A	G	2.78693E-05	0.04729	0.0207	0.0375	C4:0

UBE2E2	S27_45305602	27	45305602	ss1850288766	Intronic	G	T	0.000390674	0.08504	0.0149	0.0155	C13:0
	S27_45432002	27	45432002	ss1850288809	Intronic	C	A	7.26399E-05	0.04781	0.0186	0.0279	C6:0
	S27_45432002	27	45432002	ss1850288809	Intronic	C	A	5.27747E-05	0.06014	0.0193	0.0279	C8:0
GLUD1	S28_41433808	28	41433808	rs475314514	Intronic	T	C	5.56226E-07	0.00283	0.0294	0.0302	C4:0
	S28_41433849	28	41433849	rs466502048	Intronic	C	T	0.000165101	0.05104	0.0168	0.0155	C13:0
	S28_41435026	28	41435026	ss1850295086	Intronic	A	G	2.94546E-05	0.06479	0.0206	0.0173	C4:0
KIRREL3	S29_31425184	29	31425184	ss1850299507	Intronic	G	T	7.55195E-07	0.00209	0.03	0.0191	C24:0
	S29_31631591	29	31631591	ss1850299552	Intronic	C	A	3.29265E-05	0.0275	0.0204	0.0276	C6:0
	S29_31631591	29	31631591	ss1850299552	Intronic	C	A	0.000129458	0.09691	0.0173	0.0276	C8:0
	S29_31663455	29	31663455	ss1850299569	Intronic	C	A	0.000133213	0.03512	0.0173	0.02	C13:0
MACROD1	S29_43911453	29	43911453	rs132922154	Intronic	C	A	0.000458916	0.09173	0.0145	0.0167	C13:0
	S29_43949399	29	43949399	ss1850302054	Intronic	A	G	2.83479E-05	0.02488	0.0207	0.0233	C6:0
	S29_43949399	29	43949399	ss1850302054	Intronic	A	G	9.67741E-05	0.0821	0.018	0.0233	C8:0
	S29_43950350	29	43950350	rs451632156	Intronic	T	C	4.39636E-08	0.00026	0.0351	0.0161	C6:0
	S29_43950350	29	43950350	rs451632156	Intronic	T	C	1.85899E-07	0.00129	0.0319	0.0161	C8:0

Table S9: One SNP significantly associated with two or more fatty acid traits

Marker	Ch	Position	RS# or SS#	Classification	Gene(s)	Minor Allele	Major Allele	Proportion of Variance Explained	Minor Allele Frequency	Fatty acid
S4_20307923	4	20307923	rs462192019	Intronic	THSD7A	G	A	0.0199	0.0393	C6:0, C8:0
S4_79360395	4	79360395	rs469668684	Intronic	CAMK2B	G	T	0.0322	0.0179	C6:0, C8:0
S4_120539350	4	120539350	ss1850020621	Intronic	PAXIP1	C	A	0.0241	0.0187	C6:0, C8:0
S4_123299193	4	123299193	ss1850022099	Intronic	VIPR2	C	T	0.0197	0.0162	C6:0, C8:0
S5_31462407	5	31462407	rs462777283	Intronic	TFCP2	G	T	0.021	0.0161	C6:0, C8:0
S6_121138698	6	121138698	ss1850048597	Intronic	ACOX3	G	T	0.0395	0.0197	C4:0, C6:0, C8:0
S7_50817144	7	50817144	ss1850058473	Intronic	HBEGF	C	A	0.0235	0.0409	C6:0, C8:0
S8_104441473	8	104441473	ss1850074571	Intronic	PALM2	A	C	0.0331	0.0192	C6:0, C8:0
S8_116299522	8	116299522	ss1850076308	Intronic	COLEC11	C	A	0.025	0.0173	C6:0, C8:0
S10_81311811	10	81311811	rs211170312	Intronic	TMEM229B	A	G	0.0252	0.0372	C6:0, C8:0
S11_103096195	11	103096195	ss1850113566	Intronic	PPP2R4	C	A	0.0198	0.0184	**C4:0,** C6:0, C8:0
S13_21485963	13	21485963	ss1850129455	Intronic	NEBL	G	T	0.0178	0.0167	C6:0, C8:0
S13_30840582	13	30840582	rs451505068	Downstream	RSU1	G	T	0.0225	0.019	C6:0, C8:0
S13_78331772	13	78331772	ss1850140320	Coding	KCNB1	G	T	0.0171	0.0178	C6:0, C8:0
S19_24964715	19	24964715	rs458879791	Intronic	GGT6	G	T	0.0246	0.0154	**C4:0,** C6:0, C8:0
S19_44174585	19	44174585	ss1850207539	Coding	NBR1	C	A	0.0164	0.0161	C6:0, C8:0
S19_44672003	19	44672003	ss1850207675	Upstream	SOST	G	C	0.0184	0.0207	C6:0, C8:0
S21_65116987	21	65116987	rs381368835	Upstream	EVL	T	G	0.0142	0.0242	C6:0, C8:0
S22_51831131	22	51831131	rs473131453	Intronic	SLC25A20	G	T	0.0263	0.0179	C6:0, C8:0
S22_55449849	22	55449849	rs110927574	Intronic	GHRL	G	A	0.032	0.0184	C6:0, C8:0
S22_56478689	22	56478689	ss1850242913	Intronic	ATG7	G	T	0.0189	0.019	C6:0, C8:0
S23_26203835	23	26203835	ss1850249533	Coding	CFB	G	A	0.0274	0.0186	C6:0, C8:0
S23_43066407	23	43066407	ss1850251288	Intronic	PHACTR1	C	A	0.0353	0.0204	C6:0, C8:0
S24_63724092	24	63724092	rs447857210	Intronic	BCL2	C	A	0.0334	0.0161	**C4:0,** C6:0, C8:0
S25_7154373	25	7154373	ss1850266283	Intronic	RBFOX1	C	A	0.0172	0.0447	C6:0, C8:0
S27_45432002	27	45432002	ss1850288809	Intronic	UBE2E2	C	A	0.0207	0.0279	C6:0, C8:0

S29_31631591	29	31631591	ss1850299552	Intronic	KIRREL3	C	A	0.0214	0.0276	C6:0, C8:0
S29_43949399	29	43949399	ss1850302054	Intronic	MACROD1	A	G	0.0207	0.0233	C6:0, C8:0
S29_43950350	29	43950350	rs451632156	Intronic	MACROD1	T	C	0.0351	0.0161	C6:0, C8:0

P values from 8.58E-15 to 0.0005, P value BH FDR from 7E-10 to 0.079. Detailed results on p values are listed in supplementary tables

Table S10: GBS reference pipeline options used for analysis

Plugin	Option	Value	Description
QseqToTagCountPlugin	-s	300000000	Maximum number of good reads per lane. Default: 200000000
FastqToTagCountPlugin	-s	300000000	Maximum number of good reads per lane. Default: 200000000
QseqToTagCountPlugin	-c	1	Minimum number of times a tag must be present to be output. Default: 1
FastqToTagCountPlugin	-c	1	Minimum number of times a tag must be present to be output. Default: 1
MergeMultipleTagCountPlugin	-c	5	Minimum number of times a tag must be present to be output. Default: 1
QseqToTBTPlugin	-c	1	Minimum taxa count within a qseq file for a tag to be output. Default: 1
QseqToTBTPlugin	-s	300000000	Max good reads per lane. (Optional. Default is 200000000).
QseqToTBTPlugin	-y	-y	Output in TBTByte format (counts from 0-127) instead of TBTBit (0 or 1).
FastqToTBTPlugin	-c	1	Minimum taxa count within a fastq file for a tag to be output. Default: 1
FastqToTBTPlugin	-s	300000000	Max good reads per lane. (Optional. Default is 200000000).
FastqToTBTPlugin	-y	-y	Output in TBTByte format (counts from 0-127) instead of TBTBit (0 or 1).
MergeTagsByTaxaFilesPlugin	-s	300000000	Maximum number of tags the TBT can hold while merging (default:200000000). Reduce this only if you run out of memory (omit the commas).
MergeTagsByTaxaFilesPlugin	-x		Merges tag counts of taxa with identical names if set to -x. Not performed by default
TagsToSNPByAlignmentPlugin	-mnMAF	0.01	Minimum minor allele frequency. Defaults to 0.01. SNPs that pass either the specificed minimum minor allele frequency (mnMAF) or count (mnMAC) will be output.
TagsToSNPByAlignmentPlugin	-mnLCov	0.1	Minimum locus coverage; i.e.; the proportion of taxa with at least one tag at the locus. Default: 0.1
TagsToSNPByAlignmentPlugin	-mnF		Minimum value of F (inbreeding coefficient). Not tested by default.
TagsToSNPByAlignmentPlugin	-mnMAC	10	Minimum minor allele count. Defaults to 10. SNPs that pass either the specificed minimum minor allele count (mnMAC) or frequency (mnMAF) will be output.

TagsToSNPByAlignmentPlugin	-inclRare		Include the rare alleles at sites (3 or 4th states) (default:false).
TagsToSNPByAlignmentPlugin	-mxSites	1000000	The maximum number of SNPs per chromosome for hapmap files (default = 200000)
MergeDuplicateSNPsPlugin	-callHets	-callHets	When two genotypes at a replicate SNP disagree for a taxon; call it a heterozygote. Defaults to false (=set to missing).
MergeDuplicateSNPsPlugin	-misMat	0.05	Threshold mismatch rate above which the duplicate SNPs won't be merged. Default: 0.05.
MergeIdenticalTaxaPlugin	-hetFreq	0.8	cutoff frequency between het vs. homozygote calls (default = 0.8)
tbt2vcfPlugin	-ak	3	Maximum number of alleles that are kept for each marker across the popuation; default: 3
tbt2vcfPlugin	-mnMAF	0.01	Minimum minor allele frequency (default: 0.0)
tbt2vcfPlugin	-mnLCov	0.1	Minimum locus coverage (proportion of Taxa with a genotype) (default: 0.0)
MergeDuplicateSNP_vcf_Plugin	-ak	3	Maximum number of alleles that are kept for each marker across the population; default: 3
GBSHapMapFiltersPlugin	-mnTCov	0.1	Minimum taxon coverage i.e. the minimum SNP call rate for a taxon to be included in the output; where call rate is the proportion of the SNP genotypes for a taxon that are not "N" (where N = missing). Default: 0.1
GBSHapMapFiltersPlugin	-mnSCov	0.8	Minimum site coverage i.e. the minimum call rate for a SNP to be included in the output; where call rate is the proprotion of the taxon genotypes for that SNP that are not N (where N=missing). Default: 0.1
GBSHapMapFiltersPlugin	-mnF		Minimum value of F (inbreeding coefficient). Not tested by default. DO not invoke this option unless you are working with inbred lines or an inbreeding species.
GBSHapMapFiltersPlugin	-mnMAF	0.01	Minimum minor allele frequency Default: 0.0 (no filtering);
GBSHapMapFiltersPlugin	-mxMAF	1	Maximum minor allele frequency. Default: 1.0 (no filtering);
GBSHapMapFiltersPlugin	-hLD		Specifies that samples should be filtered for high LD. Default: false (off).

End.
